# Transcutaneous vagus nerve stimulation for Parkinson’s disease: a systematic review and meta-analysis

**DOI:** 10.3389/fnagi.2024.1498176

**Published:** 2025-01-14

**Authors:** Jiatong Shan, Zehong Li, Minxiu Ji, Miao Zhang, Caidi Zhang, Yikang Zhu, Zhen Feng

**Affiliations:** ^1^Department of Psychological Medicine, Yong Loo Lin School of Medicine, National University of Singapore, Singapore, Singapore; ^2^Centre for Healthy Longevity, @AgeSingapore, National University Health System, Singapore, Singapore; ^3^New York University Shanghai, Shanghai, China; ^4^Nanchang University Queen Mary School, Nanchang, Jiangxi, China; ^5^Shanghai Mental Health Center, Shanghai Jiao Tong University School of Medicine, Shanghai, China; ^6^Shanghai Key Laboratory of Psychotic Disorders, Shanghai Mental Health Center, Shanghai Jiao Tong University School of Medicine, Shanghai, China; ^7^Affiliated Rehabilitation Hospital, Jiangxi Medical College, Nanchang University, Nanchang, Jiangxi, China

**Keywords:** transcutaneous vagus nerve stimulation, Parkinson’s disease, motor functions, cognition, meta-analysis

## Abstract

**Background:**

Transcutaneous vagus nerve stimulation (tVNS) has emerged as a novel noninvasive adjunct therapy for advanced Parkinson’s disease (PD), yet no quantitative analysis had been conducted to assess its therapeutic effect.

**Objectives:**

This review aimed to investigate the efficacy of tVNS on motor function, other potential clinical targets and its safety in various treatment conditions.

**Methods:**

We searched six databases for randomized controlled trials (RCTs) that involved treating PD patients with tVNS. Primary outcome was motor functions, including severity of motor signs, functional mobility and balance, and gait parameters. Secondary outcomes were cognition, emotion, sleep related impairments, patient reported non-motor outcomes, and any adverse events. All outcomes were classified and analyzed according to the treatment duration and medication condition of an included study. Risk of bias was evaluated by referencing Cochrane risk of bias tool 1.0. Data was analyzed by Revman 5.4.

**Results:**

6 RCTs with 176 PD patient were included. Several motor functions and non-motor functions measured during on-medication condition (severity of motor signs −0.48 [95% CI −0.93, −0.04], gait −0.48 [95% CI −0.85, −0.1], patients reported non-motor outcomes −0.4 [95% CI −0.78, −0.03]), improved significantly. However, verbal fluency, sleep-related impairment, and fatigue were negatively impacted by tVNS during on-medication condition. No distinct adverse events were reported.

**Conclusion:**

tVNS is a relatively safe adjunct treatment for PD. It has small to moderate therapeutic effects on motor functions and may negatively impact on a few other outcomes. Quality level of the evidence is low and further research is warranted.

**Systematic review registration:**

https://www.crd.york.ac.uk/prospero/#recordDetails, identifier CRD42024503322 (PROSPERO).

## 1 Introduction

Parkinson’s disease (PD) is a central motor syndrome associated with neurodegeneration in the substantia nigra pars compacta and accumulation of synuclein proteins (Ben-Shlomo et al., 2024). It is the second most prevalent neurodegenerative disease (Getz and Levin, 2017). From 1990 to 2016 globally, age-standardized prevalence rates of PD raised by 21.7%, while an increase of 74.3% of its crude prevalence rate was witnessed (GBD 2016 Parkinson's Disease Collaborators, 2018). At the cellular level, disruptions in mitochondrial, lysosomal, and endosomal activities are evident in both monogenic and sporadic PD (Morris et al., 2024). The main symptoms of PD include tremors, bradykinesia (slowness of movement), muscular rigidity, and postural instability (DeMaagd and Philip, 2015; Chen et al., 2016).

Even as the “standard” treatment for PD, dopamine replacement medication has achieved limited progress. Therapeutic window of patients relying on it narrow by time, and eventually motor syndromes will develop, notably levodopa-induced dyskinesias (Del Sorbo and Albanese, 2008). Furthermore, the use of pharmacological treatments in PD patients may result in neurobehavioral side effects, such as hypersexuality, due to altered dopaminergic neurotransmission (Aparicio-López et al., 2024) Thus, in response to the pressing need for developing new interventions for PD, targeting the vagus nerve (VN), a non-pharmacological approach, was proposed due to its physiological role and potential therapeutic correlation with PD.

VN, the tenth cranial nerve, consists of about 80% afferent fibers and 20% efferent fibers (Bonaz et al., 2018). The nucleus of the solitary tract primarily receives and processes vagal sensory signals from the body. The dorsal motor nucleus of the vagus and nucleus ambiguus send vagal motor signals back, which is crucial for parasympathetic control (Benarroch, 1993). Furthermore, some second-order neurons of nucleus of the solitary tract project to structures including brainstem reticular formation, locus coeruleus, amygdala, periaqueductal gray, multiple raphe nuclei, parabrachial nuclei, hypothalamus, thalamus, insular cortex, etc. (Ottaviani and Macefield, 2022). By and large, VN significantly impacts the brain by enhancing motor and non-motor neural plasticity, modulating cholinergic, adrenergic and serotonergic system (Keute and Gharabaghi, 2021), regulating the release of neurotrophins (Rosso et al., 2020), and exerting anti-inflammatory effects (Bonaz et al., 2013).

Based on the innervation and function of VN, a significant overlap between the vagal-associated structures and PD-affected regions can be noticed. According to a post-mortem study and the theory that *α*-synuclein spreads in the nervous system, the proteinaceous aggregates have the potential to cause detriment to extensive parts of the brain. Susceptibility of neurons in these parts to PD and proximity to the predominantly affected regions (brainstem, limbic system, gut) may portend a risk of neurodegeneration in other areas (Braak et al., 2003; Morris et al., 2024). Neuronal loss and degeneration can therefore happen in locus coeruleus, nucleus basalis of Meynert, substantia nigra, pedunculopontine nucleus, raphe nucleus, dorsal motor nucleus of the vagus, amygdala, hypothalamus, cortices, etc. (Kalia and Lang, 2015). In addition, peripheral autonomic dysfunctions related to VN including constipation, pupillary unrest, and orthostatic hypertension, are experienced in PD patients (Sharabi et al., 2021). What’s more, VN itself is affected by PD. In PD patients, high-resolution ultrasonography studies revealed bilateral degeneration of vagus nerve (Pelz et al., 2018; Walter et al., 2018), and postmortem evidence demonstrates the vulnerability of vagal nuclei (Butt et al., 2020). PD animal models have shown varied efficacy of VNS (Farrand et al., 2017; Farrand et al., 2019; Farrand et al., 2020; Kin et al., 2021; Wang et al., 2022; Hosomoto et al., 2023), though few studies reported invasive vagus nerve stimulation (VNS) on PD patients.

Outside the context of PD, VNS is a widely applied FDA-approved approach for refractory partial onset seizures, treatment-resistant depression, obesity, and migraines (Goggins et al., 2022). In the recent past, tVNS, stemming from VNS, has gained momentum and interest in the medical field. tVNS exerts similar effects as VNS, considerably activating nucleus of the solitary tract, locus coeruleus, amygdala, hippocampus, prefrontal cortex (PFC), and other regions (Safi et al., 2016; Yakunina et al., 2017; Wang et al., 2021). Moreover, it eschews surgeries and potential risks (Carreno and Frazer, 2016; Mertens et al., 2018).

Currently, two methods comprise tVNS. The more applied one is transcutaneous auricular vagus nerve stimulation (taVNS), targeting the auricular branch of the vagus nerve (ABVN), which primarily spread through cymba conchae, cavity of conchae, tragus, and antihelix. The other technique is transcutaneous cervical vagus nerve stimulation (tcVNS). It has already been approved by FDA for treatment of refractory migraine and cluster headache (Fang et al., 2023). Akin to VNS, tcVNS stimulates the ensheathed cervical vagal branch via electrodes attached over the sternocleidomastoid muscle, usually conducted by a handheld device (Yap et al., 2020).

Motor function is prioritized in the efficacy of tVNS for PD. A recent study on priority setting partnership (PSP) for PD indicated that effective physiotherapy targeting motor function emerged as the top interest for patients and health care professionals. It showed that 79.1% of participants endorsed this focus (Bowring et al., 2022). Additionally, given the limited long-term benefits provided by pharmacological treatments and potential exacerbation of other PD symptoms, our research aligns with this patient-centric goal. Besides, considerable evidence from healthy subjects and PD patients has verified that gait is influenced by emotional and cognitive aspects, especially during complex walking conditions (Avanzino et al., 2018). Regarding emotion, studies pointed out that interoceptive state of subjects is improved by taVNS, as shown in studies concerning anxiety, stress and sleep (Aranberri Ruiz, 2024). In PD, anxiety, depression, and fatigue are prominent emotional disorders. In terms of cognition, tVNS is likely to refine executive function in PD patient as well. Healthy subjects exhibited enhancement of the ability to switch working memory states between maintenance and updating information, away from distraction, in which case taVNS was administered during the task performance (Konjusha et al., 2023).

However, gaps exist in literatures. Several studies found no significant difference in severity of motor signs between taVNS group and sham-controlled group (Yu, 2021; Lench et al., 2023; Zhang et al., 2023), whereas Mondal et al. noticed significant improvements (Mondal et al., 2023). Also, the best site of administration for taVNS remains a pending issue due to lack of detailed cutaneous mapping of ABVN and conclusive experimental evidence (Badran et al., 2018a; Burger and Verkuil, 2018; Butt et al., 2020). Apart from this, whether right VNS or left VNS is preferred needs further clarification (Brougher et al., 2021; Wang et al., 2022). Alongside disputes about tVNS, as far as we are aware, no quantitative review has been conducted to assess the efficacy of tVNS for PD, nor any adverse events (AE) in PD particularly.

Here, as the primary outcome, this review aims to meta-analyze the efficacy of tVNS for PD on motor function, including severity of motor signs, gait, and functional mobility and balance. Secondary outcomes are to assess cognition, emotion, sleep-related impairment, patient-reported non-motor outcomes, and AE. This review also aims to evaluate taVNS against tcVNS, contrast left tVNS with right tVNS, examine short-term and long-term therapeutic effects and respective AEs, and compare ON-medication and OFF-medication conditions and respective AEs. This meta-analysis will provide insights into clinical practices and further research on PD.

## 2 Methods

### 2.1 Search strategy and study selection

According to the PRISMA 2020 version for systematic reviews, the initial identification, several rounds of screening and final inclusion were conducted. In terms of databases, randomized controlled trials in electronic databases including Medline (PubMed), Embase, Cochrane Library (central), WANFANG DATA, Chinese National Knowledge Infrastructure (CNKI), Chinese Science and Technology Periodical Database (VIP) were searched from inception to April 1, 2024. The following key words were used: “Transcutaneous Vagus Nerve Stimulation,” “Transcutaneous Auricular Vagus Nerve Stimulation,” “Transcutaneous Cervical Vagus Nerve Stimulation,” “noninvasive,” and “Parkinson’s Disease.” The review was also conducted in Chinese with the following search terms: “Jing Pi,” “Fei Qin Ru,” “Wu Chuang,” “Mi Zou,” “Pa Jin Sen.” The detailed search strategies of all the databases were included in the appendix.

After removing the duplicate records in either English or Chinese databases, two authors (JS and ZL) independently screened eligible titles and abstracts. There are no restrictions on the language of publication. The references of all eligible studies were hand-searched to identify potential studies and reviews. Based on the previous inclusion or exclusion criteria, the two authors independently read the full-text articles and evaluated them. Disagreements were resolved by consensus or by consulting with other review authors and senior researchers. In addition, some methodologies in the protocol were modified in the following ways: 1. Some outcomes were excluded, including step time variability, gastrointestinal symptom rating scale, and brain activity, for they are not representative of PD. 2. Only RCTs were included to reduce heterogeneity between studies and enhance comparability and credibility of findings. 3. The language of studies were not restricted. 4. A subgroup analysis was conducted regarding study designs (parallel vs. cross-over) because in the included studies, there was no significant difference between two groups in terms of age, disease duration, etc.

### 2.2 Inclusion and extraction criteria

The studies were included if they met the following criteria: (1) were RCTs (either parallel or cross-over) (2) investigated at least one pre-defined outcomes associated with the impact of tVNS on PD patients (included but were not limited to motor functions, swallowing abilities and/or cognition); (3) selected middle-aged and elderly individuals with a primary diagnosis of PD clinically; (4) tVNS was performed in the intervention group; (5) sham stimulation was performed in the control group; (6) were not review papers.

**Figure 1:**
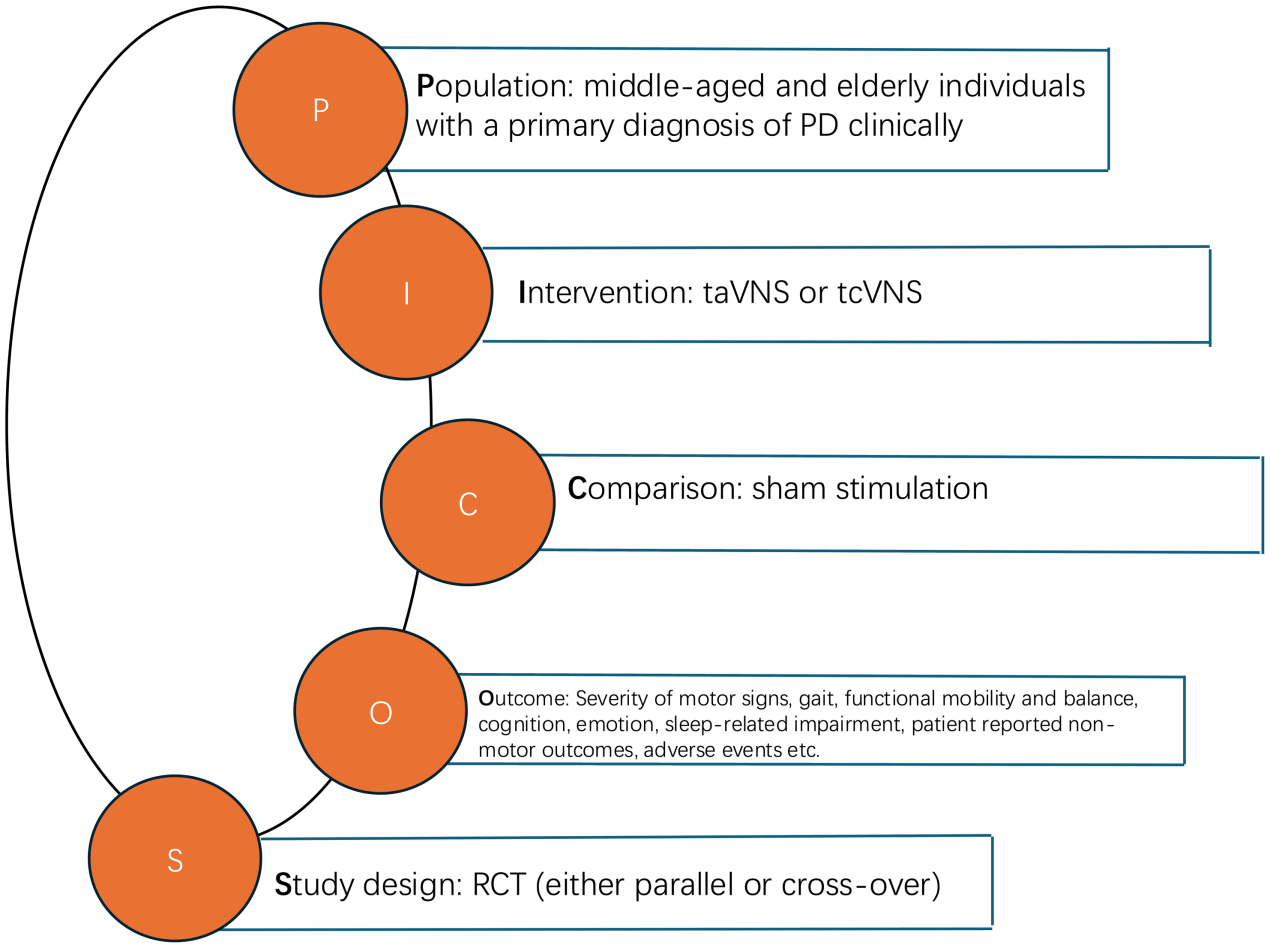
PICOS pattern.

Studies were excluded if they (1) did not include PD patients; (2) did not investigate outcomes related to clinically approved outcomes of PD; (3) were retracted; (4) did not have full texts; (5) were review papers; (6) did not have usable data. The pattern of the PICOS (population, intervention, comparison, outcome, study design) is indicated in Figure 1.

### 2.3 Data extraction

Two authors (JS and ZL) independently carried out data extraction and synthesis using Microsoft Excel. The following information was extracted: the name of the first author, publication year, intervention and control, sample size, experiment design, stimulation parameters, VNS placement, treatment frequency and duration (treatments were also classified into long-term if they lasted over one month and short-term if they lasted one month or less), primary outcome, secondary outcome, and medication condition (on-medication is defined as maintaining stable PD medication throughout the experiment period, while off-medication is defined as at least 12 h without PD medication before assessment). For cross-over trials, we extracted the combined data regarding two phases, as the interval is more than or equal to one week and the after-effects could be deemed avoided.

The same two authors conducted independently the risk of biases of included studies. There were several items: “random sequence generation,” “allocation concealment,” “blinding of participants and personnel,” “blinding of outcome assessment,” “incomplete outcome data,” “selective outcome reporting” and “other bias.” Each item was assigned to one of the three categories: “low risk,” “high risk,” or “unclear risk.” The judgments were based on Chapter 8 of the Cochrane Handbook for Systematic Reviews of Interventions (Higgins et al., 2019). The quality of this meta-analysis was assessed by the Grading of Recommendations Assessment, Development and Evaluation (GRADE) tool (Guyatt et al., 2008). According to the GRADE method,^1^ the level of evidence was classified into high, moderate, low, and very low for the purpose of evaluating the quality of the evidence based on the characteristics of related studies.

For articles with incomplete information and data, the first authors and corresponding authors were contacted by e-mail with at least two attempts.

### 2.4 Investigation of heterogeneity

Heterogeneity was explored by conducting two subgroup analyses based on the category: type of design (parallel-group design vs. cross-over design) and medication condition (medication intaking vs. medication withdrawal). A sensitivity analysis was conducted by leaving out some studies to check whether different outcome measurements have impact on total effect size under one category of outcomes.

### 2.5 Types of outcome measures

#### 2.5.1 Primary outcomes

**Figure 2:**
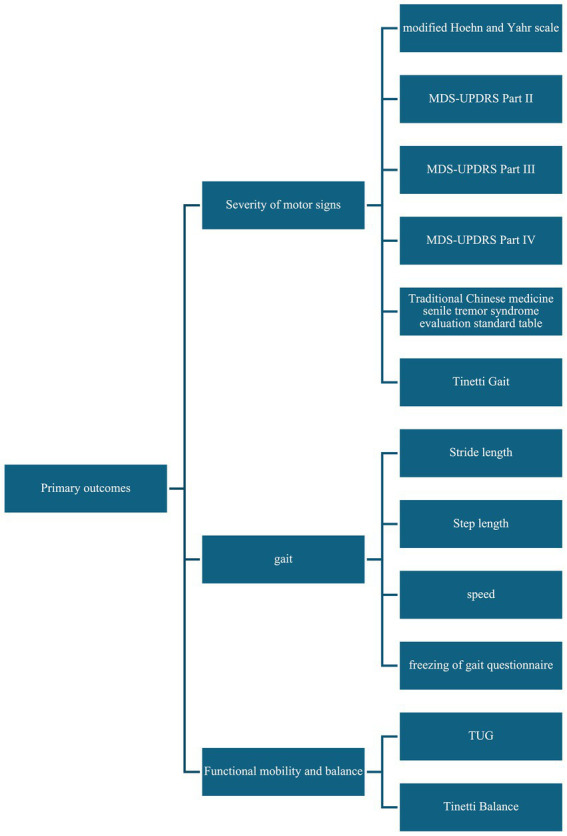
Summary of primary outcomes.

For the primary outcomes, we extracted the following data: (1) severity of motor signs (modified Hoehn and Yahr scale, MDS-UPDRS Part II, MDS-UPDRS Part III, MDS-UPDRS Part IV, Traditional Chinese medicine senile tremor syndrome evaluation standard table, and Tinetti Gait), (2) gait (stride length, step length, speed, freezing of gait questionnaire); (3) functional mobility and balance (Timed up and go (TUG) test, Tinetti Balance). Figure 2 summary of primary outcomes.

#### 2.5.2 Secondary outcomes

For the secondary outcomes, we extracted the following data: (1) cognition (PROMIS-Applied Cognition, Delis-Kaplan Executive Function System (DKEFS) letter fluency DKEFS category fluency, DKEFS category switching, Digit span forward total score, Digit span backward total score); (2) emotion; (3) sleep-related impairment; (4) patient reported non-motor outcomes, including quality of life (Parkinson’s disease questionnaire 39 (PDQ-39), MDS-UPDRS Part I, Movement Disorders Society Non-Motor Symptoms Scale for Parkinson’s Disease (NMSS), Conners Adult ADHD Rating Scale short form self-report (CAARS-S:S), fatigue) and autonomic symptoms (Scales for Outcomes in Parkinson’s Disease-Autonomic questionnaire (SCOPA- AUT)); (5) Adverse events. (6) Any outcomes in the form of change score (visit *n* to pre-screening).

### 2.6 Statistical analysis

Cochrane Revman 5.4 was utilized for data analysis. The effect size was calculated based on the sample size in active and control group, the mean and standard deviation after intervention for active tVNS and sham group. For the scales where higher scores mean better performance, positive numbers were changed into negative ones so that the more negative, the more the result favored the experimental group. For changed scores of continuous outcomes, the mean differences (MD) were estimated with 95% confidence interval (CI). If studies had endpoint outcomes, standardized mean difference (SMD) would be calculated instead of MD. Regarding binary outcomes, the risk ratio (RR) was calculated with its 95% CI. Pooled results were visualized through forest plots. To evaluate the significance of effect size, we defined it as significance if *p* ≤ 0.05, and insignificance if *p* > 0.05.

To evaluate heterogeneity, the *p* value (Cochran’s Q-test) and I^2^ statistic were utilized. If *p* > 0.1 and I^2^ < 50%, the heterogeneity was considered insignificant, and a fixed-effect model was employed for estimation. Conversely, if *p* ≤ 0.1 or I^2^ ≥ 50%, the heterogeneity was considered significant, and a random-effects model along with sensitivity analysis was conducted. The potential heterogeneity between crossover designs and parallel designs was conducted.

Registration: this systematic review was registered in PROSPERO (CRD42024503322).

## 3 Results

### 3.1 Results of the search

**Figure 3:**
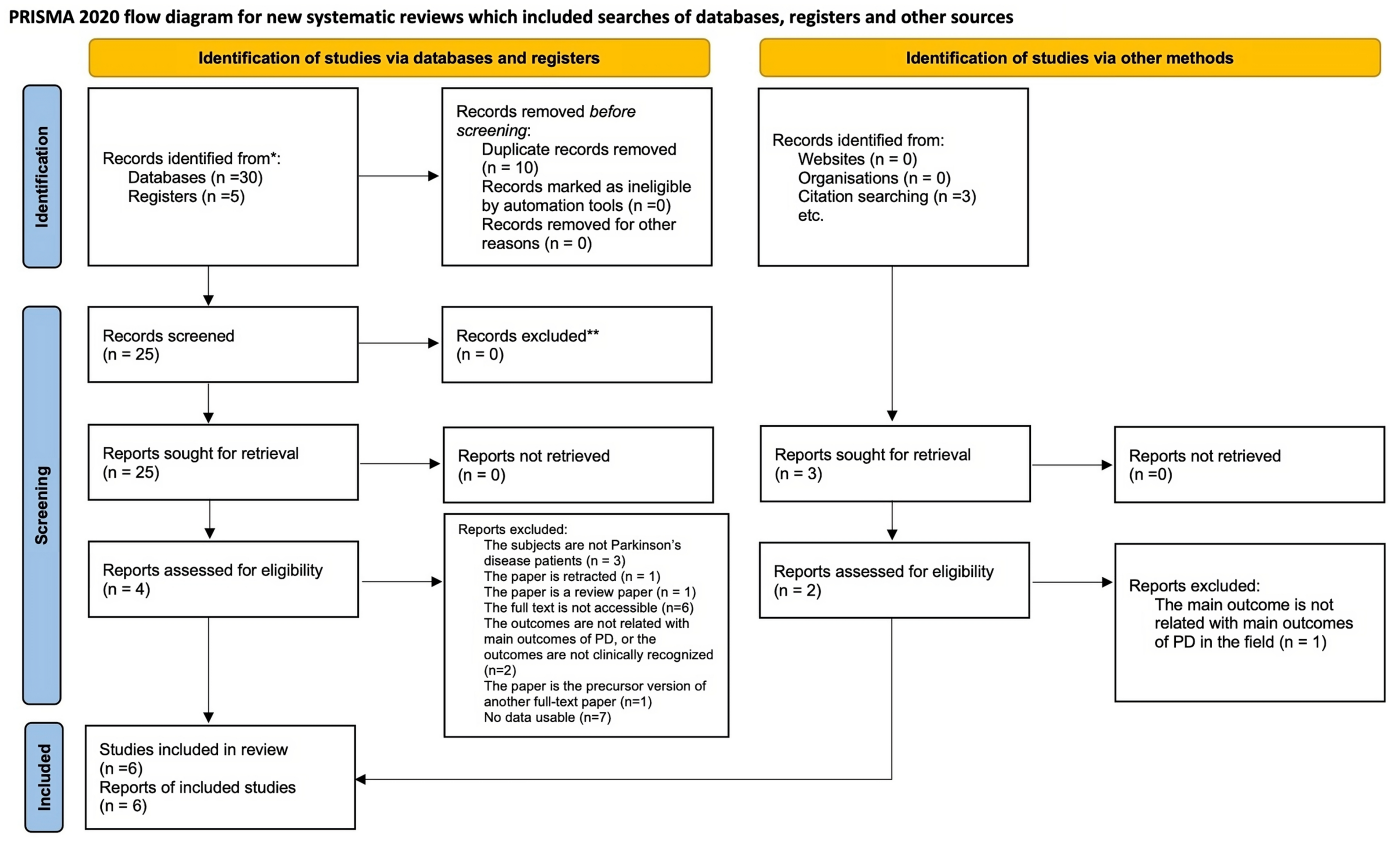
The flow of study selection.

The flow of study selection is shown in Supplementary Figure S1 (Figure 3). A total of 35 citations were identified from the databases and registers. After removing duplicate records, 25 titles and abstracts were assessed for eligibility, and 25 eligible citations remained for further full-text retrieval. After conducting a manual screening of the reference list of included articles, 3 unique citations were identified. Finally, 6 studies, which included 176 patients, fulfilled the eligibility criteria for the systematic review and meta-analysis, and the mean and standard deviation in motor function, cognition, quality of life, etc. could be obtained. Most studies applied the taVNS intervention, except for one cross-over study using tcVNS. 102 patients were in the active tVNS group, and 96 patients were in the sham activation group in this meta-analysis. Table 1 show the detailed characteristics of the included RCTs.

**Table 1:** Treatment and outcome details of the included studies.

Authors, year	Intervention and control	Sample size	Design	Stimulation parameters	VNS placement	Treatment frequency and duration	Primary outcome	Secondary outcome	Medication (on/off)	Duration (long term/short term)
Yu (2021)	taVNS and sham stimulation	29 in taVNS group and 27 in control group	Randomized and double-blind parallel study	The 20 Hz density wave and the current of 1 mA are gradually increased until the patient can tolerate without pain	The taVNS group stimulated the cymba conchae region, while the non-vagal nerve stimulation group stimulated the scapha region	8 weeks	MDS-UPDRS II&III, “Zhong Yi Lao Nian Zhen Chan Zheng Xiao Ping Ding Biao Zhun Biao”, modified Hoehn and Yahr scale	Parkinson’s disease questionnaire 39, Scales for Outcomes in Parkinsonʼs Disease-Autonomic questionnaire (SCOPA- AUT), adverse events	on	long term
Lench et al. (2023)	taVNS and sham stimulation	15 in taVNS group and 15 in control group	Randomized and double-blind parallel study	Pulse Width: 500 μs, Frequency: 25 Hz, Duty Cycle: 60s On, 30s OFF, Current Intensity: 200% perceptual threshold	Active group stimulate anterior wall of the left outer ear canal, sham stimulation group stimulation left earlobe.	1-h stimulation per day for 10 visits spread over 2 weeks	MDS-UPDRS III (acute and subacute), MDS-UPDRS I, MDS-UPDRS II, MDS-UPDRS IV	DKEFS letter fluency,DKEFS category fluency,DKEFS category switching, Digit Span Forward Total score, Digit Span Backward Total score, PROMIS fatigue, sleep Related impairment, PROMIS-Applied cognition, Conners Adult ADHD Rating Scale short form self-report (CAARS-S:S) Movement Disorders Society Non-Motor Symptoms Scale for Parkinsonʼs Disease (NMSS), adverse event	MDS-UPDRS III off, others on	short term
Marano et al. (2023)	taVNS and sham stimulation	10 PD patients with recording deep brain stimulation (DBS)	double-blind crossover trial	Frequency: 25 Hz, pulse duration: 0.3 ms	Electrodes were placed in the left external acoustic meatus at the inner side of the tragus for real taVNS and attached to the left earlobe for control stimulation	taVNS group: train duration 120 s/train, 4 trains with intervals of 60 s, while the sham group received a vibration that did not activate the vagus nerve, and after one week, all subjects were crossed over to the other.	UPDRS-III, and timed-up-and-go test (TUG),	adverse event	off	short term
Zhang et al. (2023)	taVNS and sham stimulation	22 PD patients (11 complete active,11 complete sham) and 14 HC	Randomized and double-blind parallel study	frequency: 20 Hz; pulse width: 500 μs; lasting 60 s stimulations on, alternated with 10 s off, repeat until 30 min.	Active group stimulate cymba conchae of left ear in the vicinity of the auricular branch vagus nerve, sham group also chose the same position without current.	Every PD patient received stimulation twice daily, 30 min each time, for 7 consecutive days	speed, step length, stride length, UPDRS-III, TUG, Tinetti Balance, and Tinetti Gait scores	adverse event	on	short term
Marano et al., 2022	taVNS and sham stimulation	12 patients with idiopathic PD	double-blind crossover trial	lasting 30 s each, composed of 600 pulses (frequency: 20 Hz; duration: 0.3 millisecond) repeated every 4.5 min for 30 min (six cycles)	taVNS was delivered either on the left internal tragus (real) or the earlobe (control) in trains	30 min, and then patients were randomized to one stimulation and after 1 week, all subjects were crossed over to the other.	UPDRS Part III, TUG, speed, stride length	/	on	short term
Mondal et al. (2023)	tcVNS and sham stimulation	21 participants complete sham, 25 participants complete active.	double-blind crossover trial	5 kHz sine wave stimuli of 1 ms duration at 25 Hz was produced by the active nVNS deviceat low voltage (24 V) and a maximum current output of 60 mA, Control group delivered detectable electrical stimulation to the skin (with a maximum output of 14 V and 24 mA), low frequency (0.1 Hz biphasic DC), not to activate the vagus nerve.	The stimulation was applied to the neck near the vagus nerve in the active group. The sham stimulation group stimulated the same position with low current and voltage in order not to activate the vagus nerve.	Each treatment consists of two 2-min stimulation intervals spaced 5–10 min apart, administered three times a day. The treatment lasts for one month, followed by a one-month washout period, and then patients are allocated to another treatment regimen for one month.	speed, step length, MDS-UPDRS I, MDS-UPDRS II, MDS-UPDRS III, H&Y, TUG, FOG-Q	adverse event	off	long term

### 3.2 Main results

#### 3.2.1 Primary outcomes

##### 3.2.1.1 On medication with short-term treatment

###### 3.2.1.1.1 Functional mobility and balance

Two studies (Marano et al., 2022; Zhang et al., 2023) reported the outcome of functional mobility and balance under such condition. Overall, there were no significant difference between tVNS group and sham controlled group on functional mobility and balance, and the heterogeneity of all results was not significant (*n* = 68, SMD = 0.10, 95% CI = −0.38 to 0.58, *p* = 0.69; (I^2^ = 24%, *p* = 0.27)) (Supplementary Figure S1). Specifically, for the Tinetti balance (SMD = −0.28; 95% CI = −1.12 to 0.57; *p* = 0.52) and TUG test (SMD = 0.28, 95% CI = −0.31 to 0.87, *p* = 0.35), tVNS group showed no significant difference from sham group.

###### 3.2.1.1.2 Severity of motor signs

Two studies (Marano et al., 2022; Zhang et al., 2023) measured the MDS-UPDRS III and one study (Zhang et al., 2023) measured the Tinetti gait under this scenario. Results showed that tVNS group and the control group did not exhibit a significant difference in Tinetti gait (*n* = 22, SMD = −0.53, 95% CI = −1.38 to 0.32, *p* = 0.22) or MDS-UPDRS III (*n* = 34, SMD = −0.04, 95% CI = −0.62 to 0.54, *p* = 0.89) (Supplementary Figure S2).

###### 3.2.1.1.3 Gait

**Figure 4:**
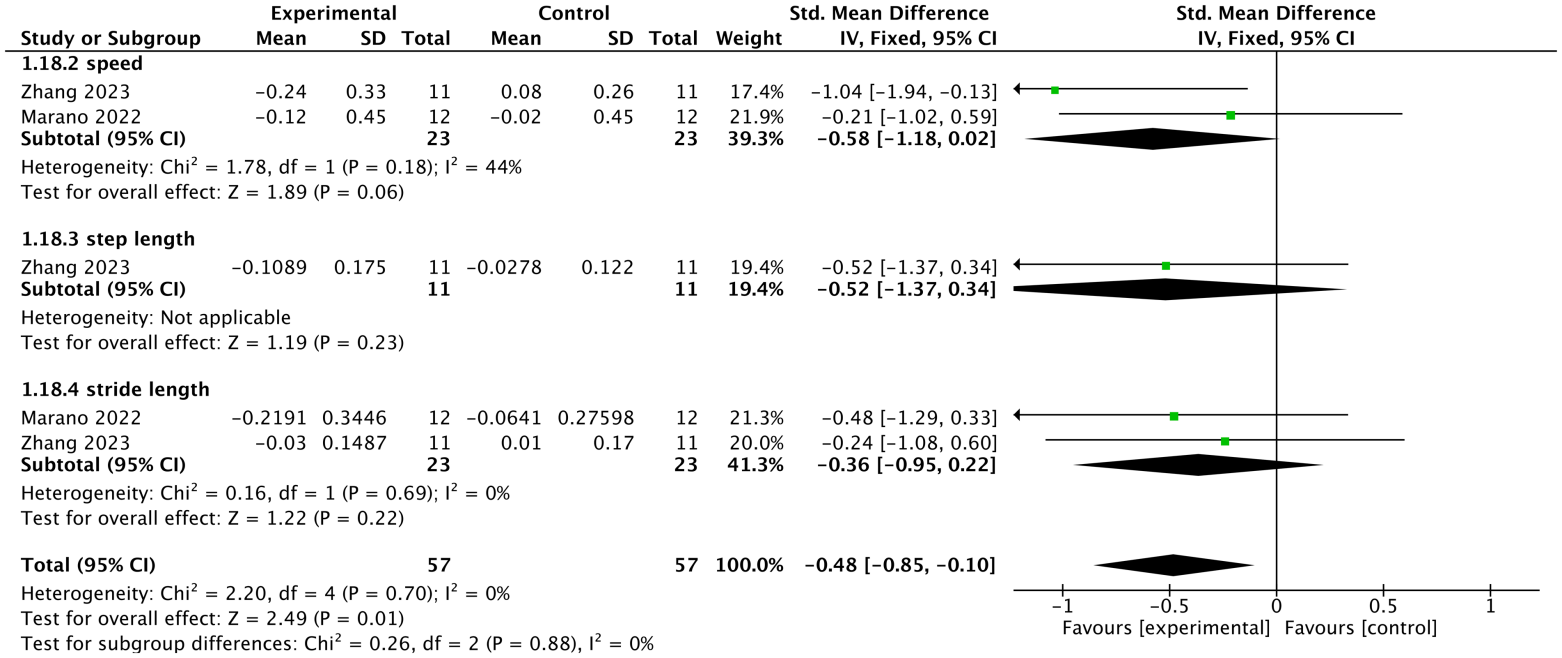
The forest plot of effects of tVNS on gait under on medication with short-term treatment.

Two studies (Marano et al., 2022; Zhang et al., 2023) reported the gait outcomes under this condition. Overall, tVNS significantly improved the gait parameters (*n* = 100, SMD = −0.48, 95% CI = −0.85 to −0.10, *p* = 0.01). tVNS also showed tendency to increase speed of PD patients compared to control group (*n* = 34, SMD = −0.58, 95% CI = −1.18 to 0.02, *p* = 0.06). However, no significant difference was identified regarding step length (SMD = −0.52, 95% CI = −1.37 to 0.34, *p* = 0.23) or stride length (SMD = −0.36, 95% CI = −0.95 to 0.22, *p* = 0.22) between tVNS group and control group. The heterogeneity was not important (I^2^ = 0%, *p* = 0.7) (Figure 4).

##### 3.2.1.2 On medication with long-term treatment

###### 3.2.1.2.1 Severity of motor signs

**Figure 5:**
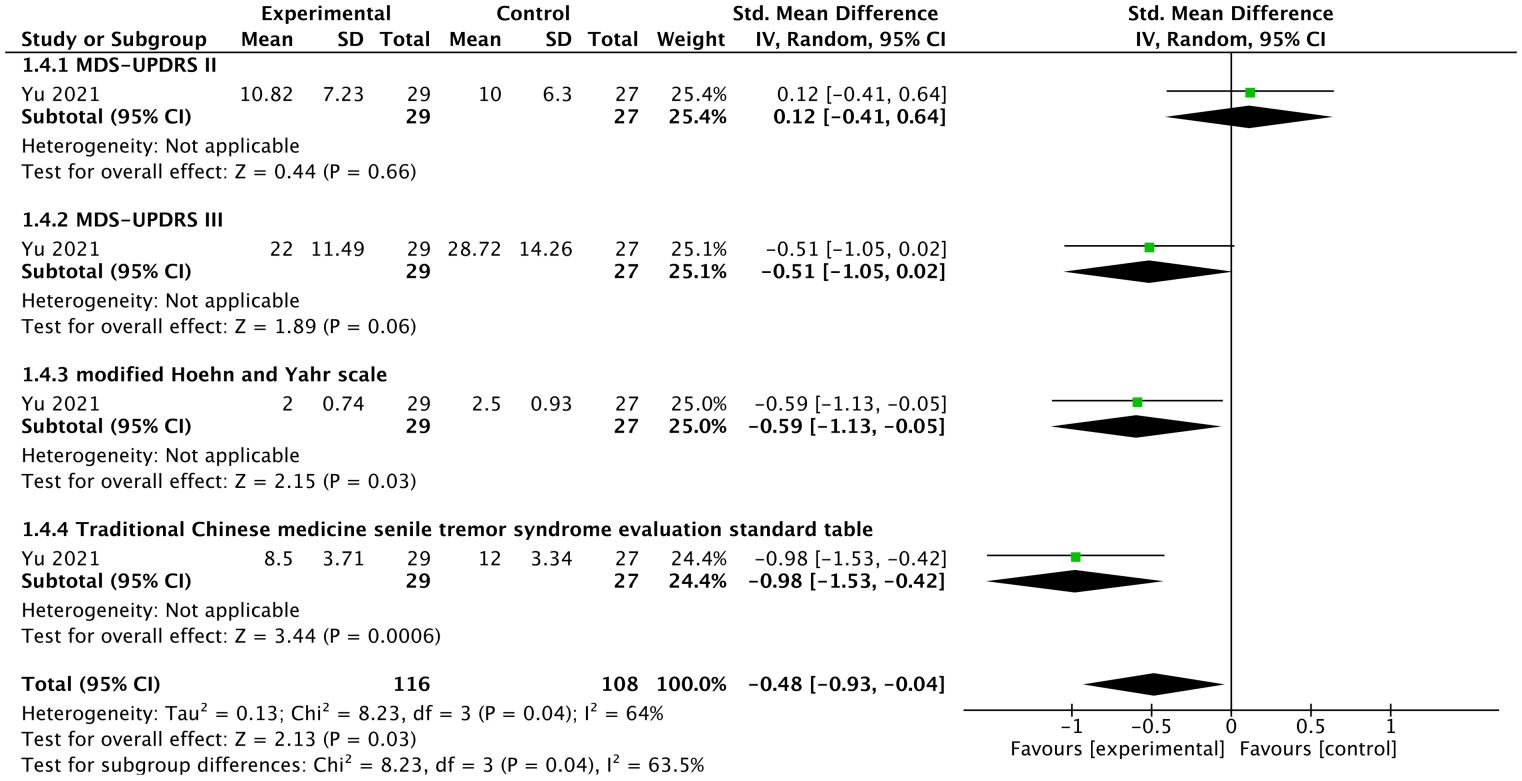
The forest plot of effects of tVNS on severity of motor signs under on medication with long-term treatment.

In one study (Yu, 2021), generally, tVNS significantly improved severity of motor signs under this combination of treatment (*n* = 224, SMD = −0.48, 95% CI = −0.93 to −0.04, *p* = 0.03) (Figure 5). There was no significant difference for MDS-UPDRS II between two groups (SMD = 0.12, 95% CI = −0.41 to 0.64, *p* = 0.66). However, a borderline improvement in MDS-UPDRS III was observed (SMD = −0.51, 95% CI = −1.05 to 0.02, *p* = 0.06). For modified Hoehn and Yahr scale (SMD = −0.59, 95% CI = −1.13 to −0.05, *p* = 0.03), and “Traditional Chinese medicine senile tremor syndrome evaluation standard table” (SMD = −0.98, 95% CI = −1.53 to −0.42, *p* = 0.0006), there were statistically significant improvement in tVNS group.

##### 3.2.1.3 Off medication with short-term treatment

###### 3.2.1.3.1 Functional mobility and balance

In one study (Marano et al., 2023), the comparison between tVNS group and control group in relation to TUG showed no significant difference (*n* = 8, SMD = −0.27; 95% CI = −4.65 to 4.11, *p* = 0.9) (Supplementary Figure S3).

###### 3.2.1.3.2 Severity of motor signs

MDS-UPDRS III acute effects, measured immediately after intervention by one study (Marano et al., 2023), did not differ significantly between tVNS group and control group (*n* = 10, SMD = 1.9, 95% CI = −15.77 to 19.57, *p* = 0.83) (Supplementary Figure S4).

##### 3.2.1.4 Off medication with long-term treatment

###### 3.2.1.4.1 Severity of motor signs

One study (Mondal et al., 2023) demonstrated that there were no significant variations between two groups in severity of motor signs (*n* = 33, SMD = 0.02, 95% CI = −0.32 to 0.35, *p* = 0.92) (Supplementary Figure S5), nor its specific items, including MDS-UPDRS III (SMD = −0.11, 95% CI = −0.69 to 0.47, *p* = 0.71), MDS-UPDRS II (SMD = 0.16, 95% CI = −0.42 to 0.75, *p* = 0.58) and modified Hoehn and Yahr scale (SMD = 0.00, 95% CI = −0.58 to 0.58, *p* = 1).

###### 3.2.1.4.2 Gait

One study (Mondal et al., 2023) investigated the gait parameters (speed, step length, FOG-Q) under such condition. It was shown that there was no significant difference for gait parameters on the whole (*n* = 33, SMD = −0.06; 95% CI = −0.4 to 0.28; *p* = 0.72) nor for speed (SMD = −0.19, 95% CI = −0.78 to 0.39, *p* = 0.51) and step length (SMD = −0.3, 95% CI = −0.89 to 0.28, *p* = 0.31) between two groups. However, tVNS may insignificantly worsen FOG-Q (SMD = 0.31, 95% CI = −0.27 to 0.9, *p* = 0.29) (Supplementary Figure S6).

#### 3.2.2 Secondary outcomes

##### 3.2.2.1 Change score

###### 3.2.2.1.1 On medication with short-term treatment

####### 3.2.2.1.1.1 Gait

One study (Lench et al., 2023) found that for the change score of FOG-Q, tVNS group may be insignificantly worsened (*n* = 29, MD = 0.7, 95% CI = −0.54 to 1.94, *p* = 0.27) (Supplementary Figure S7).

####### 3.2.2.1.1.2 Sleep-related impairment

Under this condition, one study (Lench et al., 2023) reported PROMIS sleep-related impairment, indicating that tVNS group increases the tendency of impaired sleep quality in comparison to control group (*n* = 30, MD = 4.40; 95% CI = −0.73 to 9.53, *p* = 0.09) (Supplementary Figure S8).

####### 3.2.2.1.1.3 Patients reported non-motor outcome

In one study (Lench et al., 2023), there was tendency of tVNS worsening PROMIS fatigue (*n* = 30, MD = 4.5, 95% CI = −0.23 to 9.23, *p* = 0.06). Additionally, there were no significant differences between two groups regarding MDS-UPDRS I (*n* = 26, SMD = 0.2, 95% CI = −3.03 to 3.43, *p* = 0.9), CAARS-S:S (*n* = 29, MD = 1, 95% CI = −2.71 to 4.71, *p* = 0.6), or NMSS (*n* = 26, MD = 5.3, 95% CI = −6.5 to 17.1, *p* = 0.38) (Supplementary Figure S9).

###### 3.2.2.1.2 Off medication with short-term treatment

####### 3.2.2.1.2.1 Severity of motor signs

One study (Lench et al., 2023) showed that no significant differences were identified in MDS-UPDRS II (*n* = 26, MD = −0.3, 95% CI = −2.13 to 1.53, *p* = 0.75), MDS-UPDRS III (*n* = 27, MD = 0.2, 95% CI = −3.55 to 3.95, *p* = 0.92), MDS-UPDRS IV (*n* = 25, MD = −0.7, 95% CI = −2.2 to 0.8, *p* = 0.36) between tVNS group and control group (Supplementary Figure S10).

##### 3.2.2.2 Endpoint score

###### 3.2.2.2.1 On medication with short-term treatment

####### 3.2.2.2.1.1 Cognition

**Figure 6:**
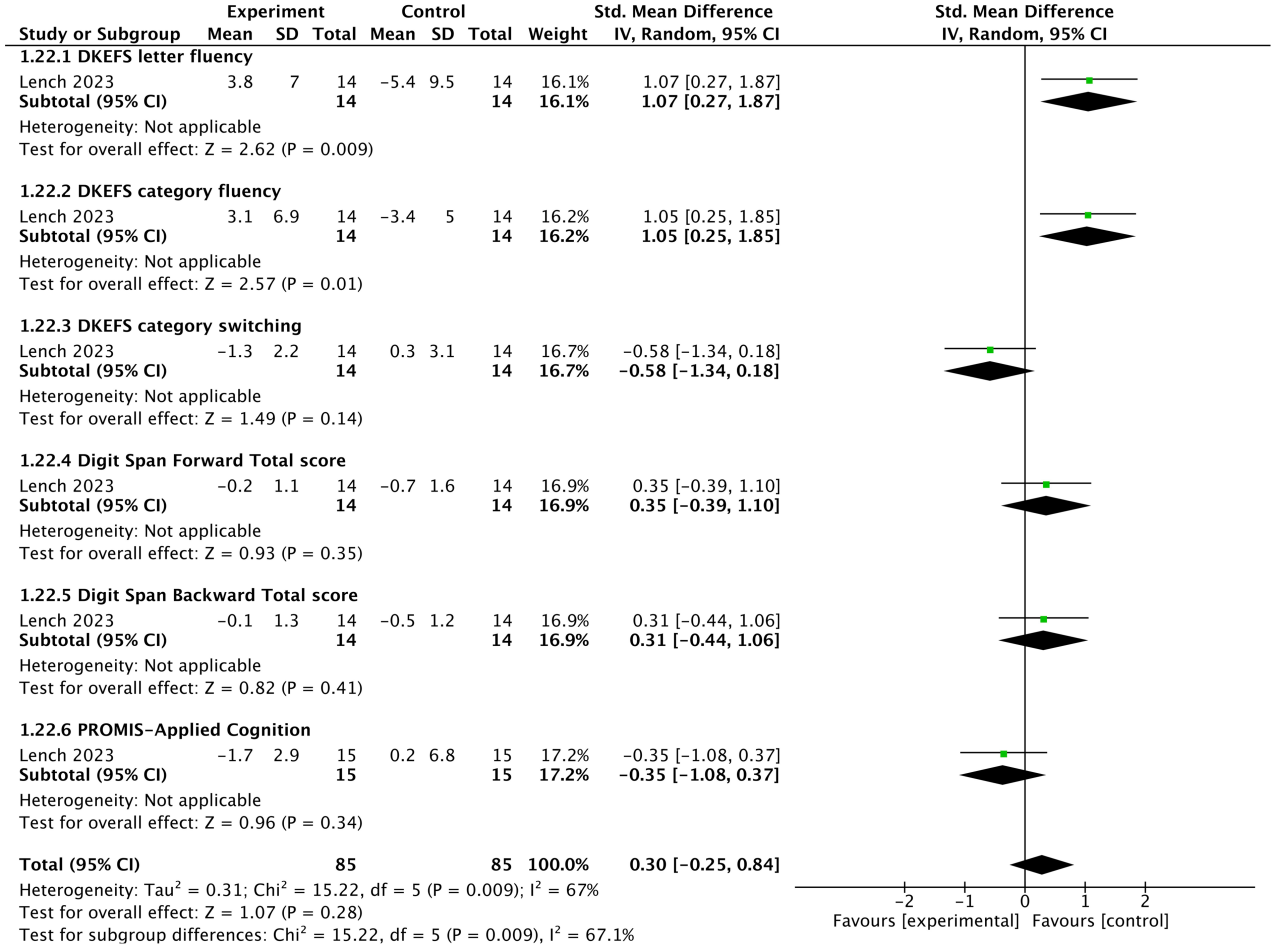
The forest plot of effects of tVNS on cognition under on medication with short-term treatment.

**Figure 7:**
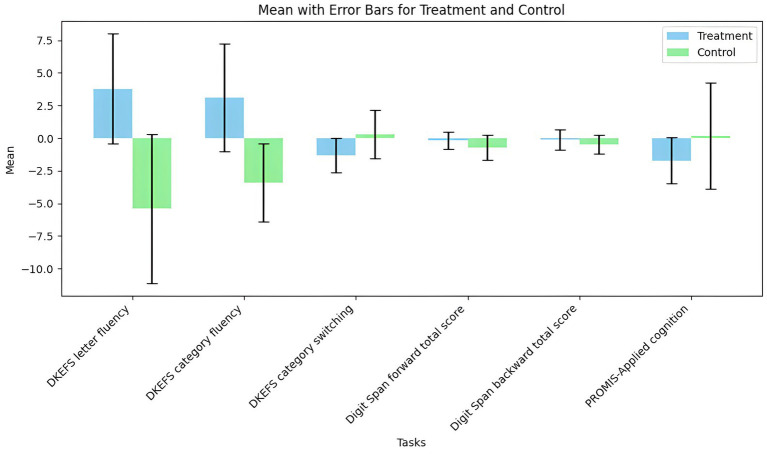
The grouped bar chart of effects of tVNS on cognition under on medication with short-term treatment.

One study (Lench et al., 2023) reported that tVNS showed an insignificantly negative impact on cognition (*n* = 170, SMD = 0.3, 95% CI = −0.25 to 0.84, *p* = 0.28). tVNS group had significantly lower scores in DKEFS letter fluency (SMD = 1.07, 95% CI = 0.27 to 1.87, *p* = 0.009) and DKEFS category fluency (SMD = 1.05, 95% CI = 0.25 to 1.85, *p* = 0.01) compared sham stimulation group. However, there were not significant differences between two groups in terms of PROMIS-Applied cognition (SMD = −0.35, 95% CI = −1.08 to 0.37, *p* = 0.34), DKEFS category switching (SMD = −0.58, 95% CI = −1.34 to 0.18, *p* = 0.14), Digit Span Forward Total Score (SMD = 0.35, 95% CI = −0.39 to 1.1, *p* = 0.35), and Digit Span Backward Total Score (SMD = 0.31, 95% CI = −0.44 to 1.06, *p* = 0.41) (Figure 6). A grouped bar chart is also provided for more intuitive visualization (Figure 7).

###### 3.2.2.2.2 On medication with long-term treatment

####### 3.2.2.2.2.1 Patients reported non-motor outcome

**Figure 8:**
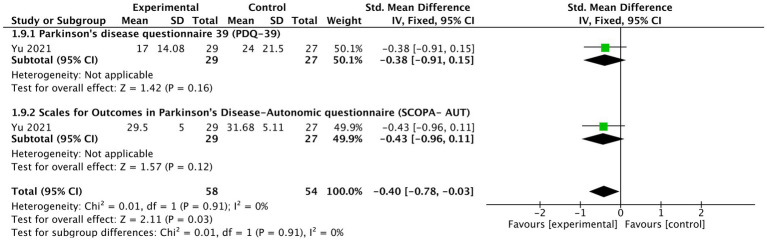
The effects of tVNS on patients reported non-motor outcome under on medication with long-term treatment.

As indicated by one study (Yu, 2021), tVNS significantly improved patient reported non-motor outcomes (*n* = 112, SMD = −0.4, 95% CI = −0.78 to −0.03, *p* = 0.03) (Figure 8). In terms of its two detailed outcomes, the tendency of improved PDQ-39 (SMD = −0.38, 95% CI = −0.91 to 0.15, *p* = 0.16) and SCOPA-AUT (SMD = −0.43, 95% CI = −0.96 to 0.11, *p* = 0.12) was noticed.

### 3.3 Adverse events (AE)

Three studies (Yu, 2021; Lench et al., 2023; Marano et al., 2023) reported AE. The frequency of AE in the active tVNS group included, vertigo 6.7% (*n* = 30), anxiousness 6.7% (*n* = 30), fluid in the ear 6.7% (*n* = 30), grinding teeth 6.7% (*n* = 30), ringing in the ear 6.7% (*n* = 30), nausea 6.7% (*n* = 30), fatigue 6.7% (*n* = 30), lightheadedness 6.7% (*n* = 30), difficulty sleeping 13.3% (*n* = 30), mild ear discomfort on the treated side 3.4% (*n* = 29), and blurred vision 1% (*n* = 10). However, there was no significant difference between two groups on AE (Supplementary Figures S11–S13). Supplementary Figures S14, S15 show the adverse events in a heatmap of experimental and control group, respectively.

### 3.4 Subgroup analysis

We conducted two subgroup analyses to assess if study design and medication condition might cause the difference between two groups. First, concerning study design, there are four outcomes that have dual occurrence in parallel designs and cross-over designs under same medication and treatment condition, with their scores being endpoints. For MDS-UPDRS III and TUG under short-term and on medication treatment, the heterogeneity was insignificant (Supplementary Figures S16, S17). However, for the outcomes of speed and stride length, the differences were significant (I^2^ = 72%, *p* = 0.06 (Supplementary Figure S18) and I^2^ = 80%, *p* = 0.03 (Supplementary Figure S19), respectively). Second, regarding medication condition, four endpoint scores overlapped between medication intake and medication withdrawal group. Medication intake did not have significant differences for TUG in short term, MDS-UPDRS II in long term and MDS-UPDRS III in long term from medication withdrawal (Supplementary Figures S20–S22). However, for the modified Hoehn and Yahr scale in the long term, the differences were significant between undertaking medication and withdrawing medication [I^2^ = 67%, *p* = 0.08 (Supplementary Figure S23)].

### 3.5 Risk of biases and level of evidence

The risk of bias was reported in Supplementary Figure S22. Two authors (JS and ZL) reached an agreement for all seven items. All studies claimed to have used randomization. Only one study (Yu, 2021) clearly showed the concealment of allocation. Two studies (Yu, 2021; Marano et al., 2023) had a high risk of blinding of participants and personnel. Three studies (Yu, 2021; Marano et al., 2023; Zhang et al., 2023) had high risk of blinding of outcome assessment, another two studies had low risk for including a blinded rater, and the other one study (Marano et al., 2022) had an unclear risk of blinding of outcome assessment because the information was unreported. Two studies (Marano et al., 2023; Mondal et al., 2023) had high risk in addressing incomplete outcome data because the reasons for losing data was likely caused by intervention, three studies (Yu, 2021; Marano et al., 2022; Zhang et al., 2023) were low-risk because either the reason for dropout was irrelevant to intervention or there were no dropouts. The reporting bias for all the studies was low-risk because the outcomes were pre-defined and related to the main outcomes for PD patients. The detailed quality of evidence is shown in Supplementary Table S1. It was based on the GRADE method and varied from moderate to very low.

## 4 Discussion

Building upon key findings presented, the discussion focuses on implications, strengths and limitations of this study. To our knowledge, this is the first meta-analysis to investigate the therapeutic efficacy of tVNS on PD patients. To recapitulate, there are several key findings in our results. First, under treatments with medication, some aspects of motor function have been ameliorated significantly. tVNS has shown significant efficacy in gait parameters, including speed, under short-term treatment; and in the severity of motor signs, including the modified Hoehn and Yahr scale and the Traditional Chinese Medicine senile tremor syndrome evaluation standard table, under long-term treatment. These improvements are reported similarly by other studies as well, although each of which does not meet our meta-analysis inclusion criteria or is a precursor of the included study (Mondal et al., 2018; Morris et al., 2019; Yarnall et al., 2019; Kumar et al., 2020; Mondal et al., 2020; Hinson et al., 2022; Marano et al., 2022; Van Midden et al., 2022; van Midden et al., 2023). These observations dovetail with progress made in animal models. In rat PD models induced by rotenone (Wang et al., 2022), 6-hydroxydopamine (6-OHDA) (Kin et al., 2021; Hosomoto et al., 2023) or combination of DSP-4 (N-(2-chloroethyl)-N-ethyl-2-bromobenzylamine) and 6-OHDA (Farrand et al., 2020), VNS consistently improves cylinder test (Farrand et al., 2020; Kin et al., 2021; Hosomoto et al., 2023), rotation test (Kin et al., 2021; Hosomoto et al., 2023), and open field test (Wang et al., 2022). It indicates that there are some restorations in motor coordination, dopaminergic system function, and general motor behavior. taVNS-fMRI studies on healthy adults also suggest increased BOLD signals in nucleus of the solitary tract and its downstream targets, including caudate, bilateral cerebellum, which are essential in motor function (Badran et al., 2018b; Borgmann et al., 2021). Nevertheless, it is noteworthy that there was worsening trend of freezing of gait in long-term treatment with medication and short-term treatment without medication. Quality assessment of included studies revealed that these results could be considered low quality of evidence due to the conspicuous advantage of the sham group in baseline (Lench et al., 2023; Mondal et al., 2023). It is still possible, however, that tVNS does not improve the subjective experiences of freezing episodes, which are influenced by cognitive load, stress, or anxiety at baseline (Witt et al., 2019). However, evidence from three studies conducted by Mondal and colleagues using video analysis of PD patients attests to efficacy of tVNS on freezing to some degree (Mondal et al., 2017; Mondal et al., 2018; Mondal et al., 2019). Notably, one of the studies identified significant improvements in freezing parameters in a long-term treatment (one month) (Mondal et al., 2017). Besides, there were no significant differences for functional mobility and balance between tVNS group and control group. Several explanations may be proposed. On the one hand, short-term treatment may not suffice to induce neuromodulation and changes in neuroplasticity for improvements in balance and mobility. On the other hand, maintaining functional mobility and balance entails coordinated processing of vestibular, visual, and proprioceptive information (Horlings et al., 2009). In PD patients, the processing of this information is altered, and tVNS may have limited influence on the restoration of vestibular nuclei, brainstem, and cerebellar regions (Silva and Israel, 2019; Lui et al., 2024). The mechanism of tVNS on motor function needs to be explored further and more clinical trials are warranted for comprehensive understanding.

Second, tVNS significantly improved the PDQ-39 during long-term treatment with medication, indicating that patients’ subjective feeling of mobility, activities of daily living, emotional well-being, social support, and communication were largely improved (Candel-Parra et al., 2021). However, it significantly worsened verbal fluency, which could probably be explained by hyperactivation of prefrontal cortex by tVNS. This phenomenon was also witnessed in deep brain stimulation in PD patients (Le Goff et al., 2015; Hinson et al., 2022). In view of improvements in verbal fluency made by tVNS in treatment-resistant depression, it may be implied that PD patients are more vulnerable to disruptions in the brain networks responsible for language and speech production (Sackeim et al., 2001). Besides, the worsening tendency of sleep-related impairment (revealed by PROMIS sleep related impairment) and patients reported non-motor outcomes (revealed by PROMIS fatigue) were identified during short-term treatment with medication. Some studies have shown that sleep quality was improved by tVNS in post-traumatic stress disorder and primary insomnia (Wu et al., 2022; Bottari et al., 2024), but few studies have focused on the treatment of tVNS on sleep in PD patients. Considering quality assessment of the included studies as well as the fact that taVNS not considerably affecting fatigue in healthy adults, more evidence is needed for determining a causal link (Yıldız et al., 2023). More attention should also be given to PD patients’ emotions during tVNS treatments including anxiety and depression.

Third, tVNS is relatively safe, indicated by no significant difference in AE between two groups, and it has many other possible implications for PD patients. To start with, tVNS may exhibit neuroprotective and anti-inflammatory effect, as showcased in animal models: There is elevated tyrosine hydroxylase level (Farrand et al., 2020; Kin et al., 2021; Wang et al., 2022; Hosomoto et al., 2023), decreased *α*-synuclein aggregation (Farrand et al., 2020; Wang et al., 2022), reduced microglial and astrocytic activation and proliferation in substantia nigra (Farrand et al., 2020; Kin et al., 2021; Hosomoto et al., 2023). Coeruleus noradrenergic neurons and substantia nigra dopaminergic neurons are protected as well (Farrand et al., 2017). In addition, tVNS relieved gastrointestinal symptoms in PD patients, although the effect is likely short-lasting (Kaut et al., 2019). From a standpoint of brain-gut axis, tVNS may as well disrupt the traverse of misfolded protein from gut to brain, though other pathogeneses are possible (Kalyanaraman et al., 2024). Furthermore, tVNS may be anxiolytic and influence depressive-like behaviors (Décarie-Spain et al., 2024). Finally, olfactory disorder in PD patients may be mitigated by tVNS (Maharjan et al., 2018).

Fourth, the optimal stimulating parameter remains uncertain. Only one study (Mondal et al., 2023) involved stimulation at the cervical level with long-term and off-medication treatment, and tcVNS insignificantly improved MDS-UPDRS III, speed, and step length. More research on tcVNS is needed to compare the efficacy between taVNS and tcVNS. Apart from this, except that only one study (Yu, 2021) stimulated both ears in a crossed way, all the other studies stimulated the left ear. Although stimulating both ears had a significant effect on motor functions, conclusions cannot yet be drawn until covariate effects such as medication and duration are adjusted. Moreover, regarding taVNS, as stimulation sites are relatively dispersed in five included studies (left external acoustic meatus, left internal tragus, cymba conchae, the anterior wall of the left outer ear canal, and left ear in the vicinity of the auricular branch vagus nerve), the optimal one is still debatable. Although most studies select 20 Hz (Zhang et al., 2023) or 25 Hz (McLeod et al., 2019; Morris et al., 2019; Hinson et al., 2022; Van Midden et al., 2022; Lench et al., 2023; Marano et al., 2023; van Midden et al., 2023) as a stimulating frequency, the optimal simulating frequency need more data to confirm.

Fifth, subgroup analysis showcased that study designs significantly caused discrepancies in speed and stride length in short-term treatment between the tVNS and sham groups while medication conditions significantly impacted the modified Hoehn and Yahr scale in long-term treatment. Because of limited numbers of included studies, further research is necessary to confirm and interpret this finding.

This review has several limitations. First, it may be underpowered due to heterogeneity of studies. The definitions of individual and stimulation parameters are different across studies. Second, results of risk of bias indicate that 50% of the included studies had more than one high risk in all judgments, which is mainly due to unknown of blinding procedure, personnel and outcome assessor, and selective reporting. Third, placebo effect may considerably affect the results. Fourth, limited RCTs available may weaken the credibility of the results. Fifth, this review does not involve some clinically important aspects, including dysphagia, aspiration, etc., which may be relieved by tVNS due to overall motor function improvement, but they deserve more attention in future tVNS research.

In general, tVNS is a relatively safe adjunct for PD therapy. It had small to moderate therapeutic effects on motor functions of PD patients. However, tVNS may impair verbal fluency, sleep quality and induce fatigue. Nevertheless, considering low quality level of outcomes due to heterogeneity and limited included studies, more randomized controlled studies with large number of subjects, focusing apart from motor functions of PD patients, are warranted for further investigations.

## 5 Conclusion

With minor to moderate therapeutic effects on motor functions, such as increasing gait speed and reducing the intensity of motor symptoms in patients receiving medication, transcutaneous vagus nerve stimulation (tVNS) may be a reasonably safe secondary treatment for Parkinson’s disease (PD), according to this review. Nevertheless, while taking medication like levodopa, tVNS was linked to some detrimental effects on verbal fluency, sleep-related problems, and weariness. Because of the small sample sizes, variability, and limited number of studies, the quality of the evidence is currently low. To better understand the therapeutic potential and safety profile of tVNS for PD, more extensive randomized controlled studies with standardized methods and more thorough outcome assessments are required in light of these limitations. Research should also look into how it affects symptoms that are not motor related. In order to optimize therapeutic advantages for individuals with Parkinson’s disease, research should also examine its impact on non-motor symptoms and the best stimulation parameters.

## Data Availability

The original contributions presented in the study are included in the article/Supplementary material, further inquiries can be directed to the corresponding authors.

## Footnotes

^1^ https://gradepro.org
