# Supplementary material for: Transcutaneous vagus nerve stimulation for Parkinson’s disease: a systematic review and meta-analysis
Source: Front Aging Neurosci. 2025 Jan 14;16:1498176. doi: 10.3389/fnagi.2024.1498176 (PMC11772336; doi:10.3389/fnagi.2024.1498176)
Supplement: Supplementary file 1 [file Data_Sheet_1.pdf]

Supplementary Material

Appendix. Search Strategies

Medline:

(TRANSCUTANEOUS VAGUS NERVE STIMULATION.ti. or TRANSCUTANEOUS VAGUS NERVE STIMULATION.ab. or TRANSCUTANEOUS AURICULAR VAGUS NERVE STIMULATION.ti. or TRANSCUTANEOUS AURICULAR VAGUS NERVE STIMULATION.ab. or TRANSCUTANEOUS CERVICAL VAGUS NERVE STIMULATION.ti. or TRANSCUTANEOUS CERVICAL VAGUS NERVE STIMULATION.ab. or TVNS.ti. or TVNS.ab. or TAVNS.ti. or TAVNS.ab. or TCVNS.ti. or TCVNS.ab. or noninvasive adj5 vagus.ti. or noninvasive adj5 vagus.ab. or noninvasive adj5 vagal.ti. or noninvasive adj5 vagal.ab. or transcutaneous adj5 vagal.ti. or transcutaneous adj5 vagal.ab.) and (randomized controlled trial.pt. or controlled clinical trial.pt. or randomized.ab. or placebo.ab. or clinical trials as topic.sh. or randomly.ab. or trial.ti. or sham.ab.) and (Parkinson Disease/ or Parkinson*.ti. or Parkinson*.ab.)

EMBASE:

(('crossover procedure':de OR 'double-blind procedure':de OR 'randomized controlled trial':de OR 'single-blind procedure':de OR (random* OR factorial* OR crossover* OR cross NEXT/1 over* OR placebo* OR doubl* NEAR/1 blind* OR singl* NEAR/1 blind* OR assign* OR allocat* OR volunteer*):de,ab,ti) OR 'crossover procedure':de OR 'double-blind procedure':de OR 'randomized controlled trial':de OR 'single-blind procedure':de) AND (Parkinson*.ti,ab. OR Parkinson Disease/exp) AND (‘TRANSCUTANEOUS VAGUS NERVE STIMULATION’/exp OR ‘TRANSCUTANEOUS AURICULAR VAGUS NERVE STIMULATION’/exp OR ‘TRANSCUTANEOUS CERVICAL VAGUS NERVE STIMULATION’/exp OR ‘TVNS’:ti,ab OR ‘TAVNS’:ti,ab OR ‘TCVNS’:ti,ab OR noninvasive NEAR/5 vag* OR transcutaneous NEAR/5 vagal)

Cochrane:

#1 TRANSCUTANEOUS VAGUS NERVE STIMULATION:TI,AB,KY

#2 TRANSCUTANEOUS AURICULAR VAGUS NERVE STIMULATION:TI,AB,KY

#3 TRANSCUTANEOUS CERVICAL VAGUS NERVE STIMULATION:TI,AB,KY

#4 TVNS:TI,AB,KY

#5 TCVNS:TI,AB,KY

#6 TAVNS:TI,AB,KY

#7 noninvasive near/5 vag*

#8 transcutaneous near/5 vagal

#9 #1 OR #2 OR #3 OR #4 OR #5 OR #6 OR #7 OR #8

#10 MeSH descriptor [Parkinson Disease] explode all trees

#11 #9 AND #10

Wanfang:

（（Subject:（ "Jing Pi"） or subject:（"Fei Qin Ru"） or subject:（"Wu Chuang"））and subject:（"Mi Zou"））and subject:（"Pa Jin Sen"）

CNKI:

（（Subject:（ "Jing Pi"） or subject:（"Fei Qin Ru"） or subject:（"Wu Chuang"））and subject:（"Mi Zou"））and subject:（"Pa Jin Sen"）

VIP:

(((M= “Jing Pi” OR “Fei Qin Ru” OR “Wu Chuang”) OR (R= “Jing Pi” OR “Fei Qin Ru” OR “Wu Chuang”)) AND (M= “Mi Zou” OR R= “Mi Zou”)) AND (M= “Pa Jin Sen” OR R= “Pa Jin Sen”)

	Experimental			Control				Std. Mean Difference
Study or Subgroup	Mean	SD	Total	Mean	SD	Total	Weight	IV, Fixed, 95% CI
**1.1.1 Tinetti balance**								
Zhang 2023	−15.64	0.67	11	−15.36	1.21	11	32.8%	−0.28 [−1.12, 0.57]
**Subtotal (95% CI)**			**11**			**11**	**32.8%**	**−0.28 [−1.12, 0.57]**
Heterogeneity: Not applicable								
Test for overall effect: Z = 0.64 (P = 0.52)								
**1.1.2 Timed Up and Go**								
Marano 2022	19.44	5.8	12	19.82	7.19	12	36.2%	−0.06 [−0.86, 0.74]
Zhang 2023	16.21	2.92	11	14.37	2.28	11	31.0%	0.68 [−0.19, 1.54]
**Subtotal (95% CI)**			**23**			**23**	**67.2%**	**0.28 [−0.31, 0.87]**
Heterogeneity: $Chi^2 = 1.48$, df = 1 (P = 0.22); $I^2 = 33\%$								
Test for overall effect: Z = 0.94 (P = 0.35)								
**Total (95% CI)**			**34**			**34**	**100.0%**	**0.10 [−0.38, 0.58]**
Heterogeneity: $Chi^2 = 2.62$, df = 2 (P = 0.27); $I^2 = 24\%$								
Test for overall effect: Z = 0.40 (P = 0.69)								
Test for subgroup differences: $Chi^2 = 1.13$, df = 1 (P = 0.29), $I^2 = 11.8\%$								

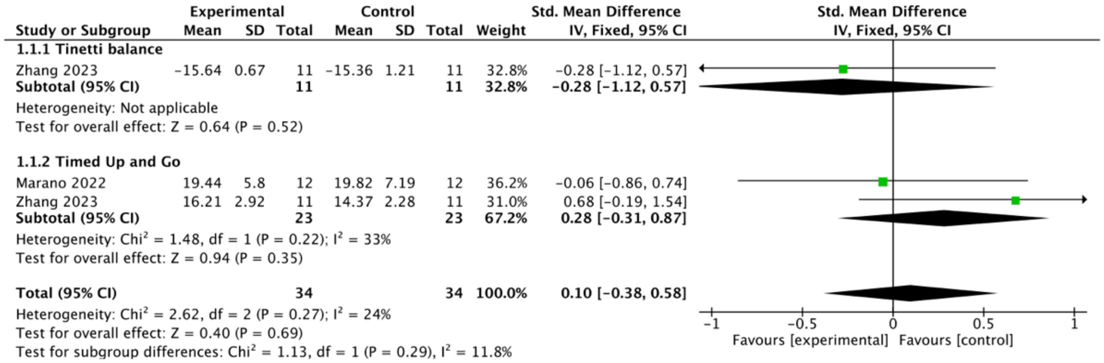

Fig.S1 The forest plot of effects of tVNS on functional mobility and balance under on medication with short-term treatment

	Experimental			Control				Std. Mean Difference
Study or Subgroup	Mean	SD	Total	Mean	SD	Total	Weight	IV, Fixed, 95% CI
**1.5.1 Tinetti gait**								
Zhang 2023	−11	0.45	11	−10.64	0.81	11	31.5%	−0.53 [−1.38, 0.32]
**Subtotal (95% CI)**			**11**			**11**	**31.5%**	**−0.53 [−1.38, 0.32]**
Heterogeneity: Not applicable								
Test for overall effect: Z = 1.21 (P = 0.22)								
**1.5.2 MDS−UPDRS III**								
Zhang 2023	13.45	6.99	11	14.27	3.35	11	32.7%	−0.14 [−0.98, 0.69]
Marano 2022	20.1	7.2	12	19.7	7.9	12	35.8%	0.05 [−0.75, 0.85]
**Subtotal (95% CI)**			**23**			**23**	**68.5%**	**−0.04 [−0.62, 0.54]**
Heterogeneity: $Chi^2 = 0.11$, df = 1 (P = 0.74); $I^2 = 0\%$								
Test for overall effect: Z = 0.14 (P = 0.89)								
**Total (95% CI)**			**34**			**34**	**100.0%**	**−0.20 [−0.67, 0.28]**
Heterogeneity: $Chi^2 = 0.96$, df = 2 (P = 0.62); $I^2 = 0\%$								
Test for overall effect: Z = 0.80 (P = 0.42)								
Test for subgroup differences: $Chi^2 = 0.86$, df = 1 (P = 0.35), $I^2 = 0\%$								

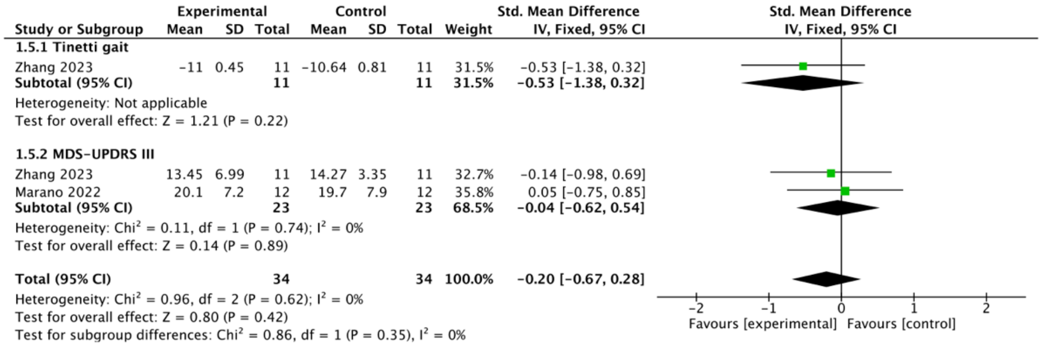

Fig.S2 The forest plot of effects of tVNS on severity of motor signs under on medication with short-term treatment

	Experimental			Control				Mean Difference
Study or Subgroup	Mean	SD	Total	Mean	SD	Total	Weight	IV, Fixed, 95% CI
**1.2.1 timed up and go**								
Marano 2023	26.63	4.86	8	26.9	4.05	8	100.0%	−0.27 [−4.65, 4.11]
**Subtotal (95% CI)**			**8**			**8**	**100.0%**	**−0.27 [−4.65, 4.11]**
Heterogeneity: Not applicable								
Test for overall effect: Z = 0.12 (P = 0.90)								
**Total (95% CI)**			**8**			**8**	**100.0%**	**−0.27 [−4.65, 4.11]**
Heterogeneity: Not applicable								
Test for overall effect: Z = 0.12 (P = 0.90)								
Test for subgroup differences: Not applicable								

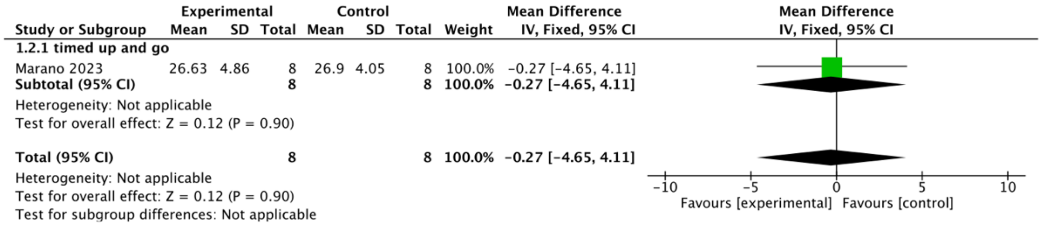

Fig.S3 The forest plot of effects of tVNS on functional mobility and balance under off medication with short-term treatment

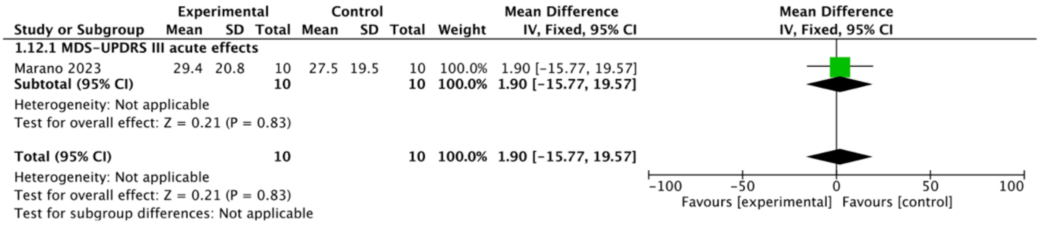

Fig.S4 The forest plot of effects of tVNS on severity of motor signs under off medication with short-term treatment

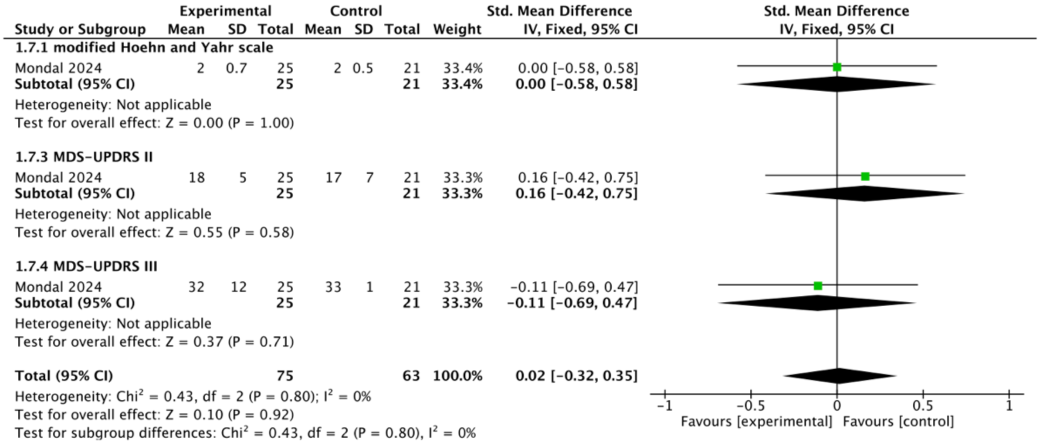

Fig.S5 The forest plot of effects of tVNS on severity of motor signs under off medication with long-term treatment

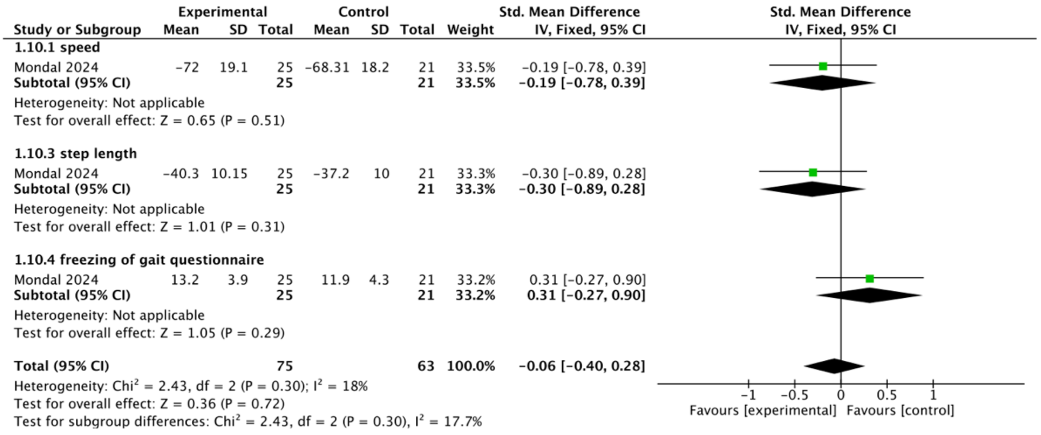

Fig.S6 The forest plot of effects of tVNS on gait under off medication with long-term treatment

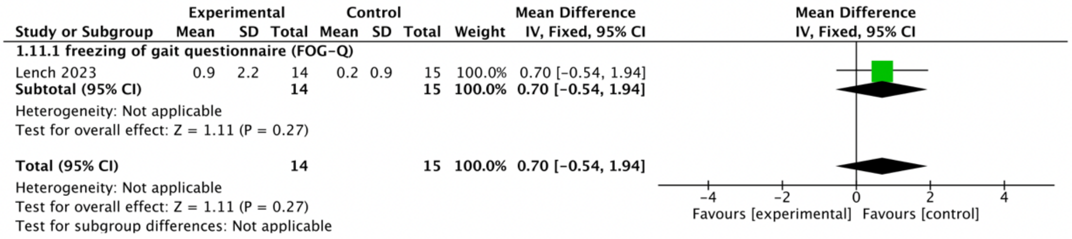

Fig. S7. Forest plot of comparison: tVNS vs sham, outcome: (secondary, change score)-gait-short term-on.

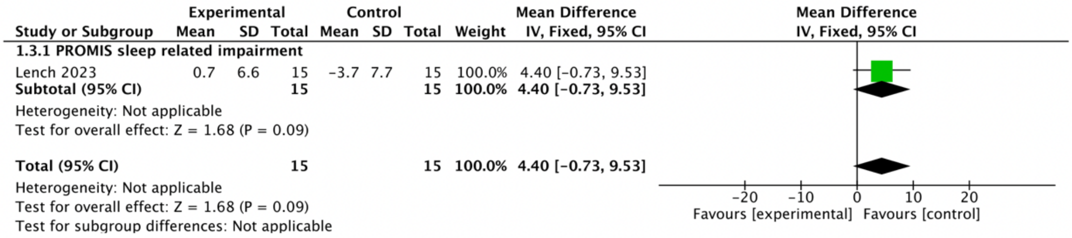

Fig. S8. Forest plot of comparison: tVNS vs sham, outcome: (secondary, change score) sleep related impairment-short term-on.

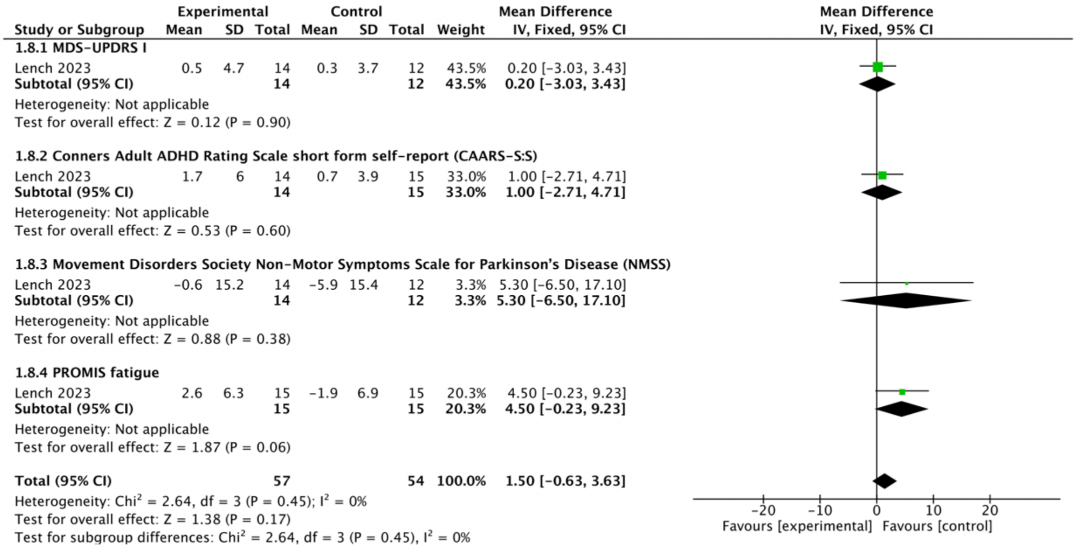

Fig. S9. Forest plot of comparison: tVNS vs sham, outcome: (secondary, change score) patients reported non-motor outcome-short term-on.

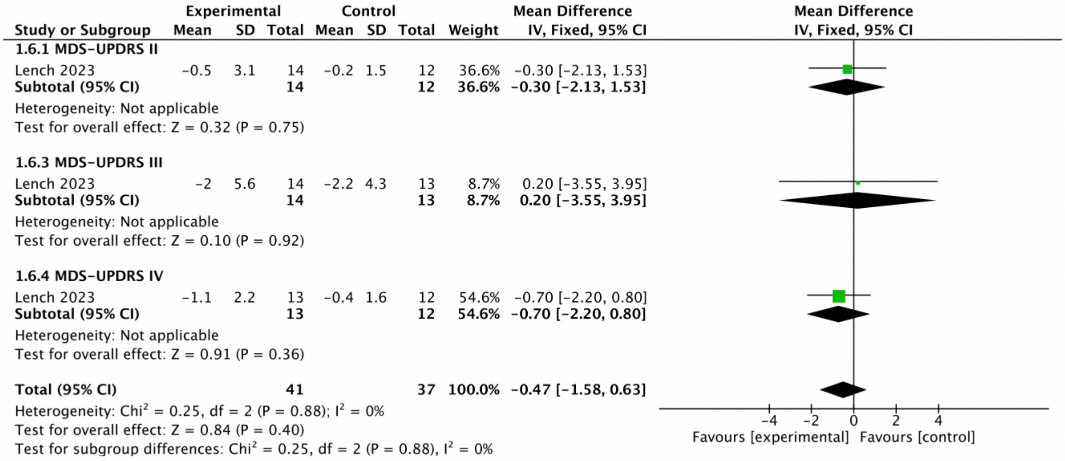

Fig. S10. Forest plot of comparison: tVNS vs sham, outcome: (secondary, change score)-severity of motor signs-short term-off.

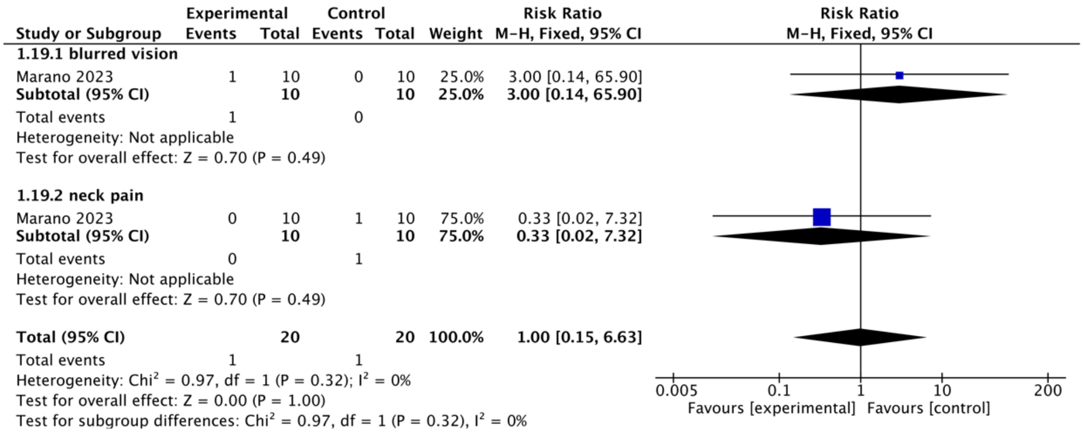

Fig. S11. Forest plot of comparison: tVNS vs sham, outcome: adverse events-short term-off.

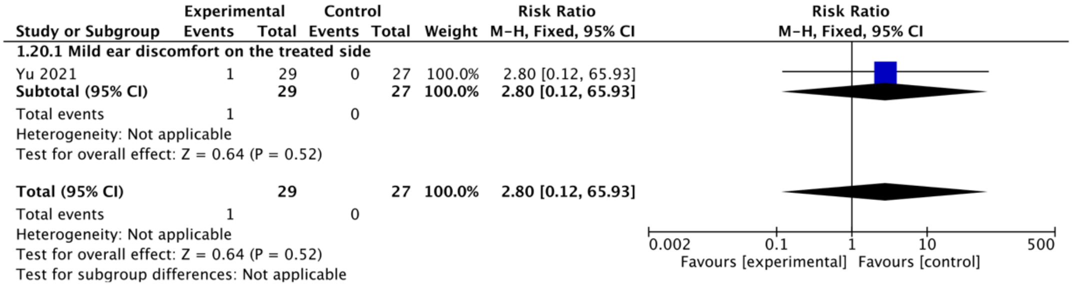

Fig. S12. Forest plot of comparison: tVNS vs sham, outcome: adverse events-long term-on.

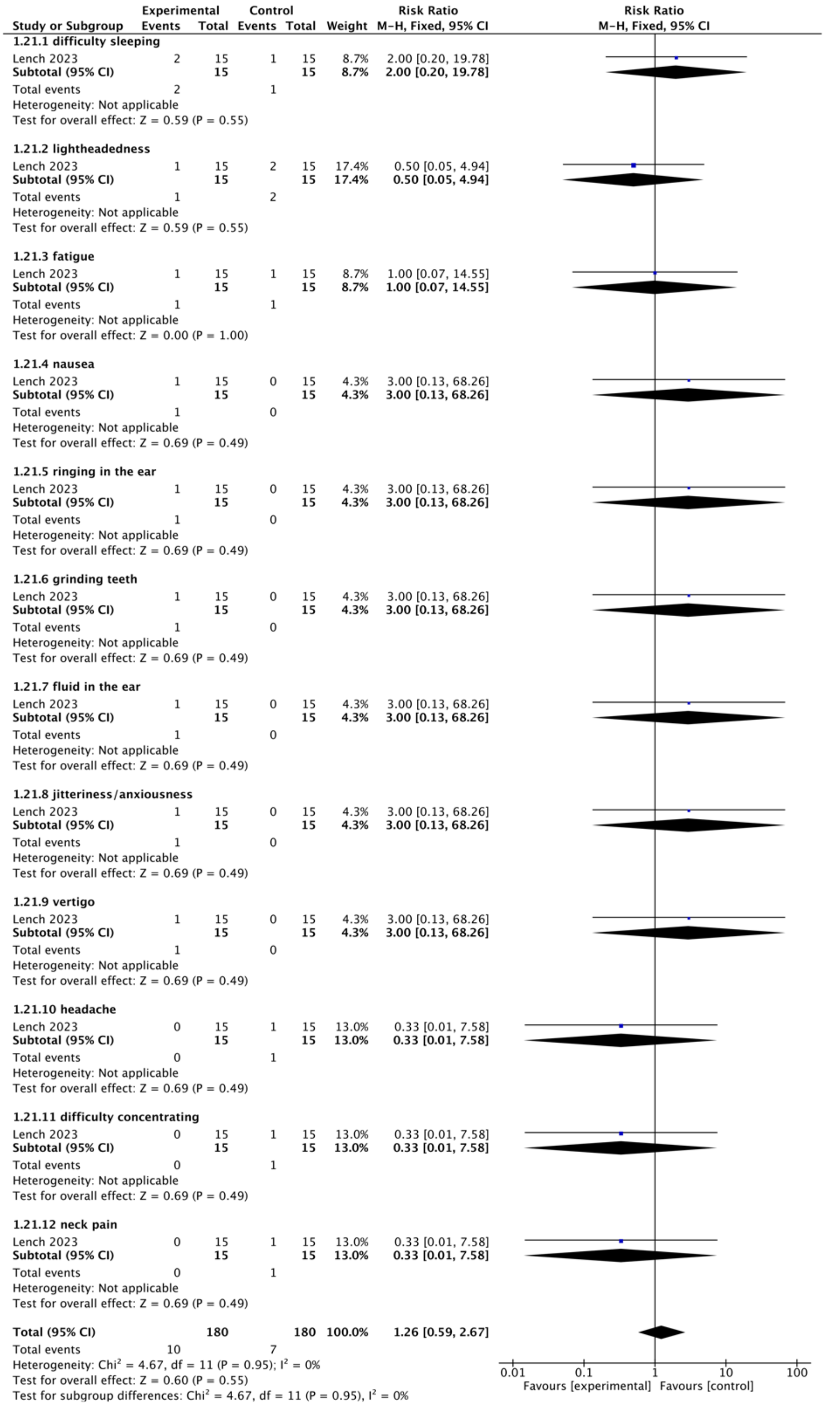

Fig. S13. Forest plot of comparison: tVNS vs sham, outcome: adverse events-short term-on.

Adverse Events of Treatment Group

	Short term and off medication	Long term and on medication	Short term and on medication
Blurred vision	1	0	0
Neck pain	0	0	0
Mild ear discomfort on the treated side	0	1	0
Difficulty sleeping	0	0	2
Lightheadedness	0	0	1
Fatigue	0	0	1
Nausea	0	0	1
Ringing in the ear	0	0	1
Grinding teeth	0	0	1
Fluid in the ear	0	0	1
Jitteriness/anxiousness	0	0	1
Vertigo	0	0	1
Headache	0	0	0
Difficulty concentrating	0	0	0

Fig. S14 Heatmap of adverse events for treatment group

Adverse Events of Control Group

	Short term and off medication	Long term and on medication	Short term and on medication
Blurred vision	0	0	0
Neck pain	1	0	1
Mild ear discomfort on the treated side	0	0	0
Difficulty sleeping	0	0	1
Lightheadedness	0	0	2
Fatigue	0	0	1
Nausea	0	0	0
Ringing in the ear	0	0	0
Grinding teeth	0	0	0
Fluid in the ear	0	0	0
Jitteriness/anxiousness	0	0	0
Vertigo	0	0	0
Headache	0	0	1
Difficulty concentrating	0	0	1

Fig. S15 Heatmap of adverse events for control group

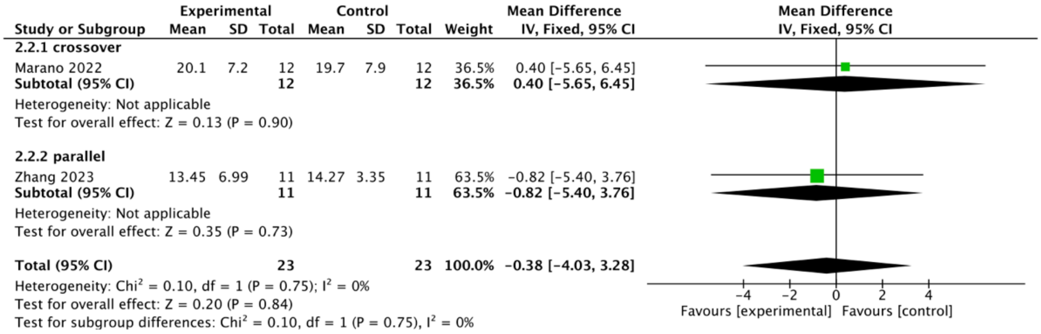

Fig. S16 Meta-analysis result of insignificant heterogeneity for outcomes of MDS-UPDRS III under different study designs

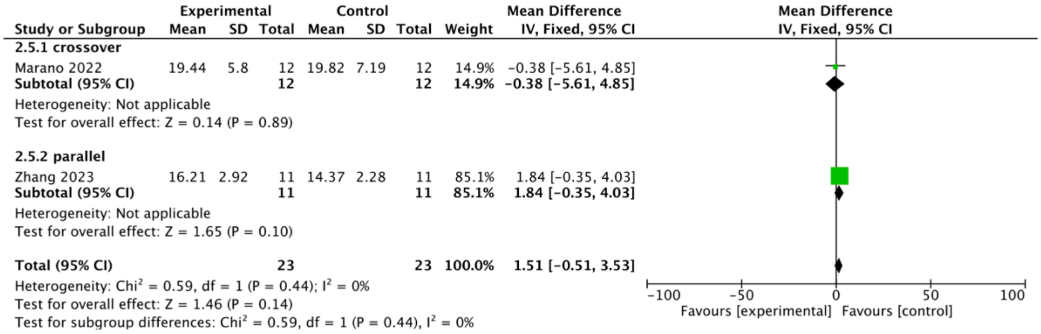

Fig.S17 Meta-analysis result of insignificant heterogeneity for outcomes of TUG under different study designs

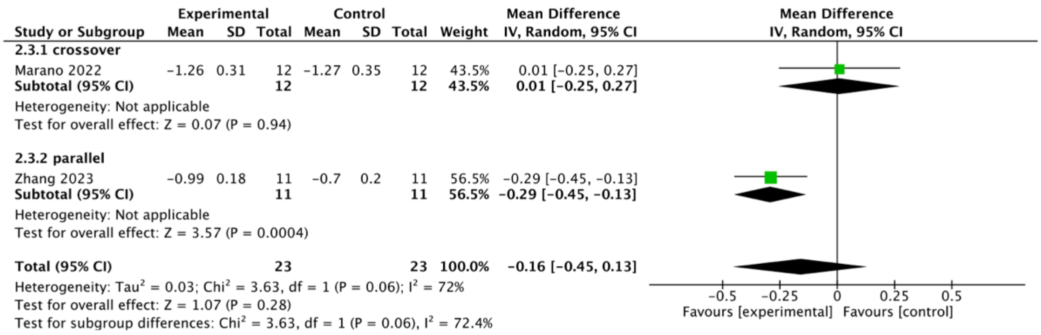

Fig.S18 Meta-analysis result of significant heterogeneity for outcomes of speed under different study designs

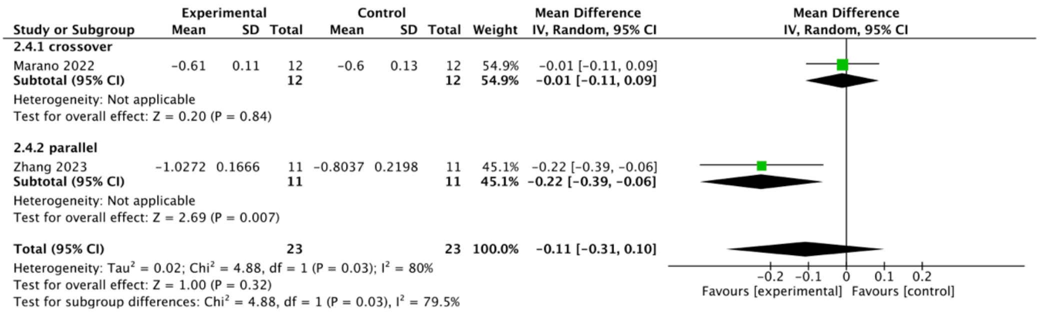

Fig.S19 Meta-analysis result of significant heterogeneity for outcomes of step length under different study designs

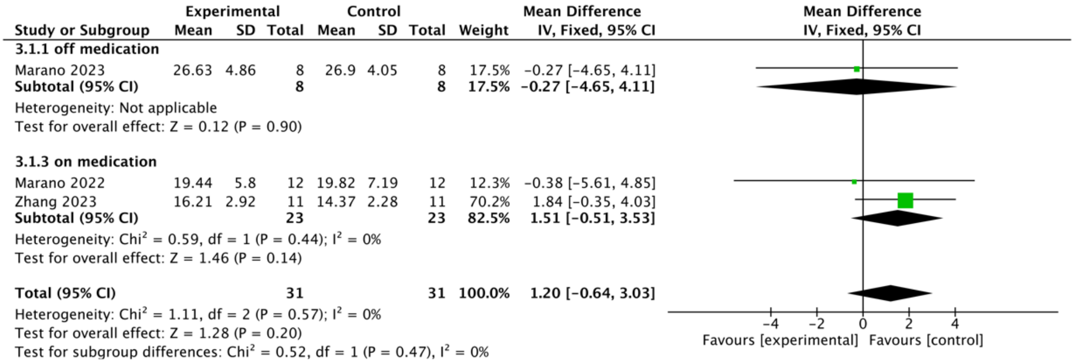

Fig.S20 Meta-analysis result of insignificant heterogeneity for outcomes of TUG under different medication conditions

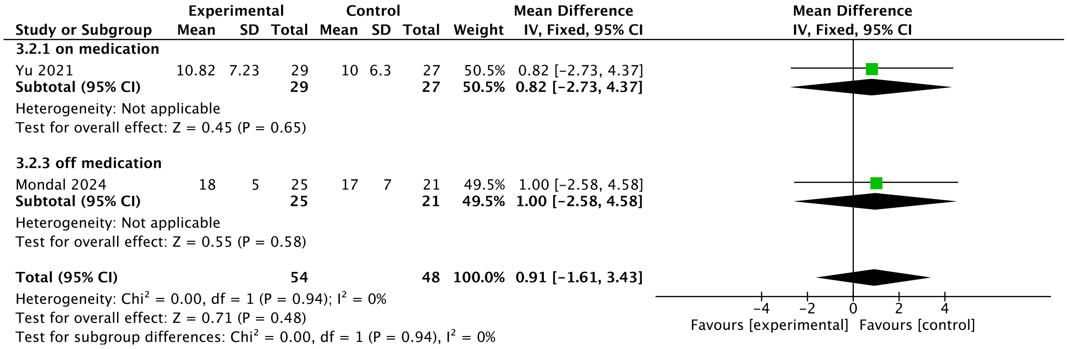

Fig.S21 Meta-analysis result of insignificant heterogeneity for outcomes of MDS-UPDRS II under different medication conditions

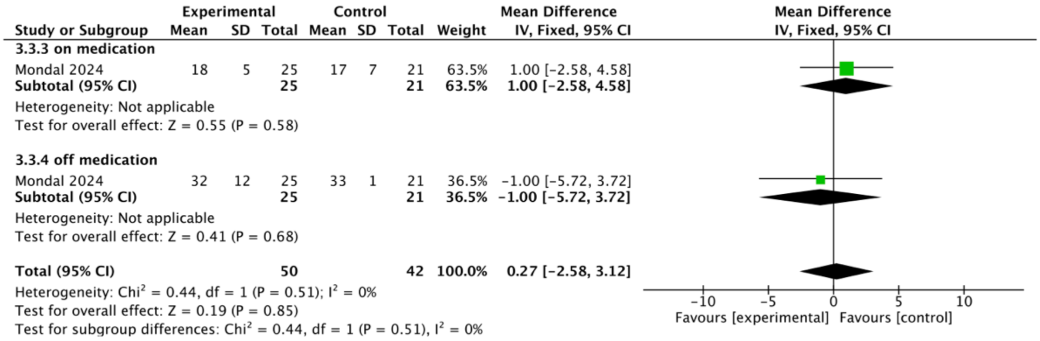

Fig.S22 Meta-analysis result of insignificant heterogeneity for outcomes of MDS-UPDRS III under different medication conditions

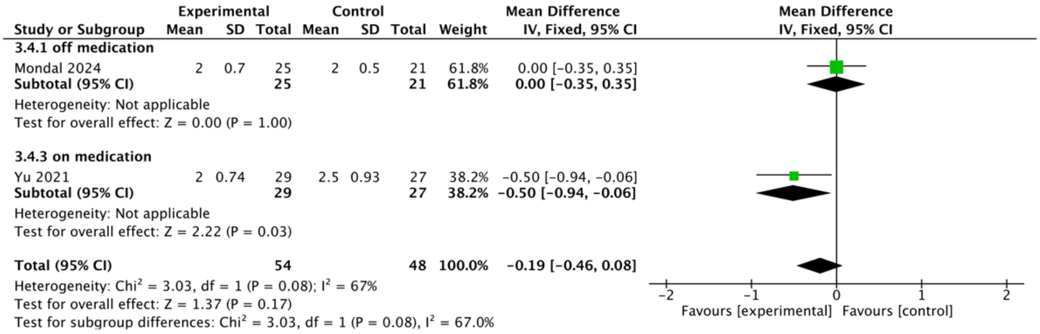

Fig.S23 Meta-analysis result of significant heterogeneity for outcomes of modified Hoehn and Yahr scale under different medication conditions

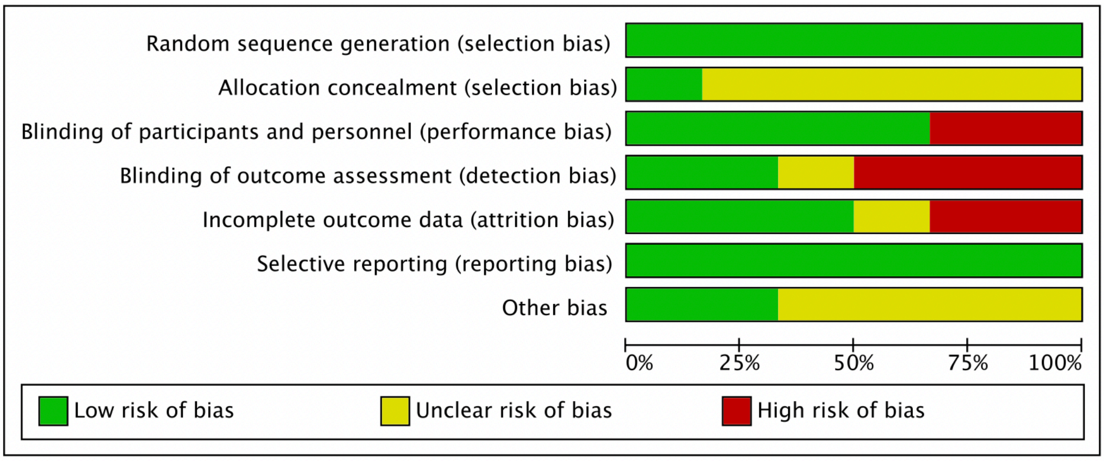

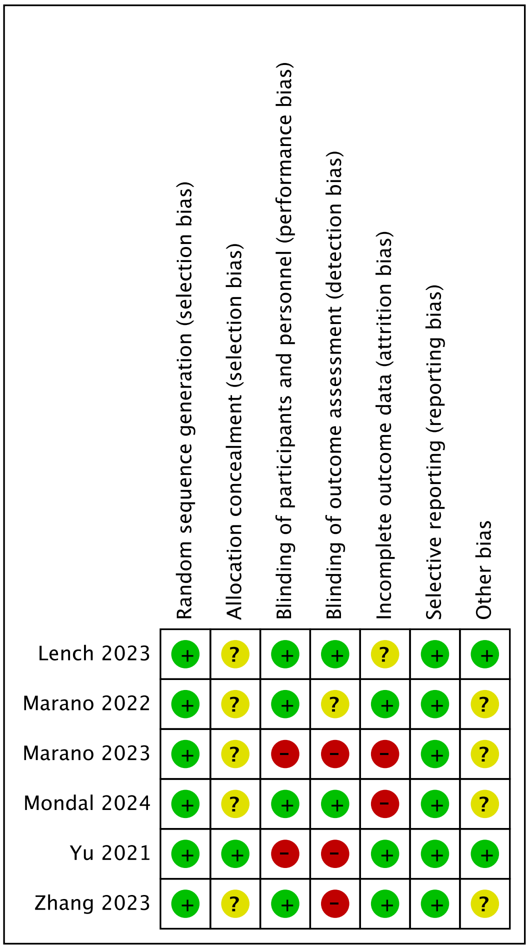

Fig. S24. Risk of bias of included studies

Table S1. Evaluation of quality of evidence

Certainty assessment							No of patients		Effect		Certainty
**No of studies**	**Study design**	**Risk of bias**	**Inconsistency**	**Indirectness**	**Imprecision**	**Other considerations**	**tVNS**	**sham**	**Relative (95% CI)**	**Absolute (95% CI)**	
**functional mobility and balance-short term-on**											
2	randomised trials	not serious	not serious	not serious	very serious[a]	none	34	34	-	SMD **0.1 higher** (0.38 lower to 0.58 higher)	⊕⊕◯◯ Low
**functional mobility and balance-short term-on - Tinetti balance**											
1	randomised trials	not serious	not serious	not serious	very serious[a]	none	11	11	-	SMD **0.28 lower** (1.12 lower to 0.57 higher)	⊕⊕◯◯ Low
**functional mobility and balance-short term-on - Timed Up and Go**											
2	randomised trials	not serious	not serious	not serious	very serious[a]	none	23	23	-	SMD **0.28 higher** (0.31 lower to 0.87 higher)	⊕⊕◯◯ Low
**functional mobility and balance-short term-off**											
1	randomised trials	not serious	not serious	not serious	very serious[a]	none	8	8	-	MD **0.27 lower** (4.65 lower to 4.11 higher)	⊕⊕◯◯ Low
**functional mobility and balance-short term-off - timed up and go**											
1	randomised trials	not serious	not serious	not serious	very serious[a]	none	8	8	-	MD **0.27 lower** (4.65 lower to 4.11 higher)	⊕⊕◯◯ Low
**(secondary, change score) sleep related impairment-short term-on**											
1	randomised trials	not serious	not serious	not serious	serious[b]	none	15	15	-	MD **4.4 higher** (0.73 lower to 9.53 higher)	⊕⊕⊕◯ Moderate
**(secondary, change score) sleep related impairment-short term-on - PROMIS sleep related impairment**											
1	randomised trials	not serious	not serious	not serious	very serious[a]	none	15	15	-	MD **4.4 higher** (0.73 lower to 9.53 higher)	⊕⊕◯◯ Low
**severity of motor signs-long term-on**											
1	randomised trials	not serious	serious[c]	not serious	not serious	none	116	108	-	SMD **0.48 lower** (0.93 lower to	⊕⊕⊕◯ Moderate

										0.04 lower)	
**severity of motor signs-long term-on - MDS-UPDRS II**											
1	randomised trials	not serious	not serious	not serious	very serious[a]	none	29	27	-	SMD **0.12 higher** (0.41 lower to 0.64 higher)	⨁⨁◯◯ Low
**severity of motor signs-long term-on - MDS-UPDRS III**											
1	randomised trials	not serious	not serious	not serious	serious[b]	none	29	27	-	SMD **0.51 lower** (1.05 lower to 0.02 higher)	⨁⨁⨁◯ Moderate
**severity of motor signs-long term-on - modified Hoehn and Yahr scale**											
1	randomised trials	not serious	not serious	not serious	serious[d]	none	29	27	-	SMD **0.59 lower** (1.13 lower to 0.05 lower)	⨁⨁⨁◯ Moderate
**severity of motor signs-long term-on - Traditional Chinese medicine senile tremor syndrome evaluation standard table**											
1	randomised trials	not serious	not serious	not serious	serious	none	29	27	-	SMD **0.98 lower** (1.53 lower to 0.42 lower)	⨁⨁⨁◯ Moderate
**severity of motor signs-short term-on**											
2	randomised trials	not serious	not serious	not serious	very serious[a]	none	34	34	-	SMD **0.2 lower** (0.67 lower to 0.28 higher)	⨁⨁◯◯ Low
**severity of motor signs-short term-on - Tinetti gait**											
1	randomised trials	not serious	not serious	not serious	very serious[a]	none	11	11	-	SMD **0.53 lower** (1.38 lower to 0.32 higher)	⨁⨁◯◯ Low
**severity of motor signs-short term-on - MDS-UPDRS III**											
2	randomised trials	not serious	not serious	not serious	very serious[a]	none	23	23	-	SMD **0.04 lower** (0.62 lower to 0.54 higher)	⨁⨁◯◯ Low
**(secondary, change score) severity of motor signs-short term-off**											
1	randomised trials	not serious	not serious	not serious	very serious[a]	none	55	50	-	SMD **0.04 lower** (0.42 lower to 0.35 higher)	⨁⨁◯◯ Low

**(secondary, change score) severity of motor signs-short term-off - MDS-UPDRS II**											
1	randomised trials	not serious	not serious	not serious	very serious[a]	none	14	12	-	SMD **0.12 lower** (0.89 lower to 0.66 higher)	⊕⊕◯◯ Low
**(secondary, change score) severity of motor signs-short term-off - MDS-UPDRS III**											
1	randomised trials	not serious	not serious	not serious	very serious[a]	none	14	13	-	SMD **0.04 higher** (0.72 lower to 0.79 higher)	⊕⊕◯◯ Low
**(secondary, change score) severity of motor signs-short term-off - MDS-UPDRS IV**											
1	randomised trials	not serious	not serious	not serious	very serious[a]	none	13	12	-	SMD **0.35 lower** (1.14 lower to 0.44 higher)	⊕⊕◯◯ Low
**severity of motor signs-long term-off**											
1	randomised trials	not serious	not serious	not serious	very serious[a]	none	75	63	-	SMD **0.02 higher** (0.32 lower to 0.35 higher)	⊕⊕◯◯ Low
**severity of motor signs-long term-off - modified Hoehn and Yahr scale**											
1	randomised trials	not serious	not serious	not serious	very serious[a]	none	25	21	-	SMD **0** (0.58 lower to 0.58 higher)	⊕⊕◯◯ Low
**severity of motor signs-long term-off - MDS-UPDRS II**											
1	randomised trials	not serious	not serious	not serious	very serious[a]	none	25	21	-	SMD **0.16 higher** (0.42 lower to 0.75 higher)	⊕⊕◯◯ Low
**severity of motor signs-long term-off - MDS-UPDRS III**											
1	randomised trials	not serious	not serious	not serious	very serious[a]	none	25	21	-	SMD **0.11 lower** (0.69 lower to 0.47 higher)	⊕⊕◯◯ Low
**(secondary, change score) patients reported non-motor outcome-short term-on**											
1	randomised trials	not serious	not serious	not serious	serious[b]	none	57	54	-	SMD **0.31 higher** (0.06 lower to 0.69 higher)	⊕⊕⊕◯ Moderate
**(secondary, change score) patients reported non-motor outcome-short term-on - MDS-UPDRS I**											

1	randomised trials	not serious	not serious	not serious	very serious[a]	none	14	12	-	SMD **0.05 higher** (0.73 lower to 0.82 higher)	⊕⊕◯◯ Low
**(secondary, change score) patients reported non-motor outcome-short term-on - Conners Adult ADHD Rating Scale short form self-report (CAARS-S:S)**											
1	randomised trials	not serious	not serious	not serious	very serious[a]	none	14	15	-	SMD **0.19 higher** (0.54 lower to 0.92 higher)	⊕⊕◯◯ Low
**(secondary, change score) patients reported non-motor outcome-short term-on - Movement Disorders Society Non-Motor Symptoms Scale for Parkinson's Disease (NMSS)**											
1	randomised trials	not serious	not serious	not serious	very serious[a]	none	14	12	-	SMD **0.34 higher** (0.44 lower to 1.11 higher)	⊕⊕◯◯ Low
**(secondary, change score) patients reported non-motor outcome-short term-on - PROMIS fatigue**											
1	randomised trials	not serious	not serious	not serious	very serious[a]	none	15	15	-	SMD **0.66 higher** (0.08 lower to 1.4 higher)	⊕⊕◯◯ Low
**patient reported non-motor outcome-long term-on**											
1	randomised trials	not serious	very serious[e]	not serious	very serious[a]	none	58	54	-	SMD **0.18 higher** (1.01 lower to 1.38 higher)	⊕◯◯◯ Very low
**patient reported non-motor outcome-long term-on - Parkinson's disease questionnaire 39 (PDQ-39)**											
1	randomised trials	not serious	not serious	not serious	serious[d]	none	29	27	-	SMD **0.79 higher** (0.25 higher to 1.34 higher)	⊕⊕⊕◯ Moderate
**patient reported non-motor outcome-long term-on - Scales for Outcomes in Parkinson's Disease-Autonomic questionnaire (SCOPA- AUT)**											
1	randomised trials	not serious	not serious	not serious	very serious[a]	none	29	27	-	SMD **0.43 lower** (0.96 lower to 0.11 higher)	⊕⊕◯◯ Low
**gait-long term-off**											
1	randomised trials	not serious	not serious	not serious	serious[b]	none	100	84	-	SMD **0.07 lower** (0.36 lower to 0.22 higher)	⊕⊕⊕◯ Moderate
**gait-long term-off - speed**											

1	randomised trials	not serious	not serious	not serious	very serious[a]	none	25	21	-	SMD **0.19 lower** (0.78 lower to 0.39 higher)	⊕⊕◯◯ Low
**gait-long term-off - timed up and go**											
1	randomised trials	not serious	not serious	not serious	very serious[a]	none	25	21	-	SMD **0.09 lower** (0.67 lower to 0.49 higher)	⊕⊕◯◯ Low
**gait-long term-off - step length**											
1	randomised trials	not serious	not serious	not serious	very serious[a]	none	25	21	-	SMD **0.3 lower** (0.89 lower to 0.28 higher)	⊕⊕◯◯ Low
**gait-long term-off - freezing of gait questionnaire**											
1	randomised trials	not serious	not serious	not serious	very serious[a]	none	25	21	-	SMD **0.31 higher** (0.27 lower to 0.9 higher)	⊕⊕◯◯ Low
**(secondary, change score)-gait-short term-on**											
1	randomised trials	not serious	not serious	not serious	very serious[a]	none	14	15	-	MD **0.7 higher** (0.54 lower to 1.94 higher)	⊕⊕◯◯ Low
**(secondary, change score)-gait-short term-on - freezing of gait questionnaire (FOG-Q)**											
1	randomised trials	not serious	not serious	not serious	very serious[a]	none	14	15	-	MD **0.7 higher** (0.54 lower to 1.94 higher)	⊕⊕◯◯ Low
**severity of motor signs-short term-off**											
1	randomised trials	not serious	not serious	not serious	very serious[a]	none	10	10	-	MD **1.9 higher** (15.77 lower to 19.57 higher)	⊕⊕◯◯ Low
**severity of motor signs-short term-off - MDS-UPDRS III acute effects**											
1	randomised trials	not serious	not serious	not serious	very serious[a]	none	10	10	-	MD **1.9 higher** (15.77 lower to 19.57 higher)	⊕⊕◯◯ Low
**gait-short term-on**											
2	randomised trials	not serious	not serious	not serious	serious[d]	none	57	57	-	SMD **0.48 lower** (0.85 lower to 0.1 lower)	⊕⊕⊕◯ Moderate

**gait-short term-on - speed**											
2	randomised trials	not serious	not serious	not serious	serious[b]	none	23	23	-	SMD **0.58 lower** (1.18 lower to 0.02 higher)	⊕⊕⊕◯ Moderate
**gait-short term-on - step length**											
1	randomised trials	not serious	not serious	not serious	serious[b]	none	11	11	-	SMD **0.52 lower** (1.37 lower to 0.34 higher)	⊕⊕⊕◯ Moderate
**gait-short term-on - stride length**											
2	randomised trials	not serious	not serious	not serious	serious[b]	none	23	23	-	SMD **0.36 lower** (0.95 lower to 0.22 higher)	⊕⊕⊕◯ Moderate
**adverse events-short term-off**											
1	randomised trials	not serious	not serious	not serious	very serious[a]	none	1/20 (5.0%)	1/20 (5.0%)	**RR 1.00** (0.15 to 6.63)	**0 fewer per 1,000** (from 43 fewer to 282 more)	⊕⊕◯◯ Low
**adverse events-short term-off - blurred vision**											
1	randomised trials	serious[f]	not serious	not serious	very serious[a]	none	1/10 (10.0%)	0/10 (0.0%)	**RR 3.00** (0.14 to 65.90)	**0 fewer per 1,000** (from 0 fewer to 0 fewer)	⊕◯◯◯ Very low
**adverse events-short term-off - neck pain**											
1	randomised trials	serious[f]	not serious	not serious	very serious[a]	none	0/10 (0.0%)	1/10 (10.0%)	**RR 0.33** (0.02 to 7.32)	**67 fewer per 1,000** (from 98 fewer to 632 more)	⊕◯◯◯ Very low
**adverse events-long term-on**											
1	randomised trials	not serious	not serious	not serious	very serious[a]	none	1/29 (3.4%)	0/27 (0.0%)	**RR 2.80** (0.12 to 65.93)	**0 fewer per 1,000** (from 0 fewer to 0 fewer)	⊕⊕◯◯ Low
**adverse events-long term-on - Mild ear discomfort on the treated side**											
1	randomised trials	not serious	not serious	not serious	very serious[a]	none	1/29 (3.4%)	0/27 (0.0%)	**RR 2.80** (0.12 to 65.93)	**0 fewer per 1,000** (from 0 fewer to 0 fewer)	⊕⊕◯◯ Low
**adverse events-short term-on**											
1	randomised trials	not serious	not serious	not serious	very serious[a]	none	10/180 (5.6%)	7/180 (3.9%)	**RR 1.26** (0.59 to 2.67)	**10 more per 1,000** (from 16	⊕⊕◯◯ Low

										fewer to 65 more)	
**adverse events-short term-on - difficulty sleeping**											
1	randomised trials	not serious	not serious	not serious	very serious[a]	none	2/15 (13.3%)	1/15 (6.7%)	**RR 2.00** (0.20 to 19.78)	**67 more per 1,000** (from 53 fewer to 1,000 more)	⊕⊕◯◯ Low
**adverse events-short term-on - lightheadedness**											
1	randomised trials	not serious	not serious	not serious	very serious[a]	none	1/15 (6.7%)	2/15 (13.3%)	**RR 0.50** (0.05 to 4.94)	**67 fewer per 1,000** (from 127 fewer to 525 more)	⊕⊕◯◯ Low
**adverse events-short term-on - fatigue**											
1	randomised trials	not serious	not serious	not serious	very serious[a]	none	1/15 (6.7%)	1/15 (6.7%)	**RR 1.00** (0.07 to 14.55)	**0 fewer per 1,000** (from 62 fewer to 903 more)	⊕⊕◯◯ Low
**adverse events-short term-on - nausea**											
1	randomised trials	not serious	not serious	not serious	very serious[a]	none	1/15 (6.7%)	0/15 (0.0%)	**RR 3.00** (0.13 to 68.26)	**0 fewer per 1,000** (from 0 fewer to 0 fewer)	⊕⊕◯◯ Low
**adverse events-short term-on - ringing in the ear**											
1	randomised trials	not serious	not serious	not serious	very serious[a]	none	1/15 (6.7%)	0/15 (0.0%)	**RR 3.00** (0.13 to 68.26)	**0 fewer per 1,000** (from 0 fewer to 0 fewer)	⊕⊕◯◯ Low
**adverse events-short term-on - grinding teeth**											
1	randomised trials	not serious	not serious	not serious	very serious[a]	none	1/15 (6.7%)	0/15 (0.0%)	**RR 3.00** (0.13 to 68.26)	**0 fewer per 1,000** (from 0 fewer to 0 fewer)	⊕⊕◯◯ Low
**adverse events-short term-on - fluid in the ear**											
1	randomised trials	not serious	not serious	not serious	very serious[a]	none	1/15 (6.7%)	0/15 (0.0%)	**RR 3.00** (0.13 to 68.26)	**0 fewer per 1,000** (from 0 fewer to 0 fewer)	⊕⊕◯◯ Low
**adverse events-short term-on - jitteriness/anxiousness**											
1	randomised trials	not serious	not serious	not serious	very serious[a]	none	1/15 (6.7%)	0/15 (0.0%)	**RR 3.00** (0.13 to 68.26)	**0 fewer per 1,000** (from 0 fewer to 0 fewer)	⊕⊕◯◯ Low
**adverse events-short term-on - vertigo**											

1	randomised trials	not serious	not serious	not serious	very serious[a]	none	1/15 (6.7%)	0/15 (0.0%)	**RR 3.00** (0.13 to 68.26)	**0 fewer per 1,000** (from 0 fewer to 0 fewer)	⨁⨁◯◯ Low
**adverse events-short term-on - headache**											
1	randomised trials	not serious	not serious	not serious	very serious[a]	none	0/15 (0.0%)	1/15 (6.7%)	**RR 0.33** (0.01 to 7.58)	**45 fewer per 1,000** (from 66 fewer to 439 more)	⨁⨁◯◯ Low
**adverse events-short term-on - difficulty concentrating**											
1	randomised trials	not serious	not serious	not serious	very serious[a]	none	0/15 (0.0%)	1/15 (6.7%)	**RR 0.33** (0.01 to 7.58)	**45 fewer per 1,000** (from 66 fewer to 439 more)	⨁⨁◯◯ Low
**adverse events-short term-on - neck pain**											
1	randomised trials	not serious	not serious	not serious	very serious[a]	none	0/15 (0.0%)	1/15 (6.7%)	**RR 0.33** (0.01 to 7.58)	**45 fewer per 1,000** (from 66 fewer to 439 more)	⨁⨁◯◯ Low
**cognition-short term-on**											
1	randomised trials	not serious	not serious	not serious	very serious[a]	none	85	85	-	SMD **0.04 higher** (0.26 lower to 0.34 higher)	⨁⨁◯◯ Low
**cognition-short term-on - DKEFS letter fluency**											
1	randomised trials	not serious	not serious	not serious	very serious[a]	none	14	14	-	SMD **0.19 higher** (0.56 lower to 0.93 higher)	⨁⨁◯◯ Low
**cognition-short term-on - DKEFS category fluency**											
1	randomised trials	not serious	not serious	not serious	very serious[a]	none	14	14	-	SMD **0.05 higher** (0.69 lower to 0.79 higher)	⨁⨁◯◯ Low
**cognition-short term-on - DKEFS category switching**											
1	randomised trials	not serious	not serious	not serious	very serious[a]	none	14	14	-	SMD **0.36 lower** (1.11 lower to 0.39 higher)	⨁⨁◯◯ Low
**cognition-short term-on - Digit Span Forward Total score**											
1	randomised trials	not serious	not serious	not serious	very serious[a]	none	14	14	-	SMD **0.35 higher** (0.39 lower to	⨁⨁◯◯ Low

										1.1 higher)	
**cognition-short term-on - Digit Span Backward Total score**											
1	randomised trials	not serious	not serious	not serious	very serious[a]	none	14	14	-	SMD **0.31 higher** (0.44 lower to 1.06 higher)	⊕⊕◯◯ Low
**cognition-short term-on - PROMIS-Applied Cognition**											
1	randomised trials	not serious	not serious	not serious	very serious[a]	none	15	15	-	SMD **0.28 lower** (1 lower to 0.44 higher)	⊕⊕◯◯ Low

CI: confidence interval; **MD:** mean difference; **RR:** risk ratio; **SMD:** standardised mean difference

Explanations

a. Downgraded by two levels: the optimal information size (OIS) was not met and the confidence interval included both appreciable benefit and harm

b. the confidence interval included both appreciable benefit and harm

c. I^2 statistic, which quantifies the proportion of the variation in point estimates due to among-study differences, is large

d. the optimal information size (OIS) was not met

e. I2 statistic, which quantifies the proportion of the variation in point estimates due to among-study differences, is considerable

f. The proportion of information from studies at high risk of bias is sufficient to affect the interpretation of results.
